# Ligand-Centered Triplet Diradical Supported by a Binuclear Palladium(II) Dipyrrindione

**DOI:** 10.1021/acs.inorgchem.1c01691

**Published:** 2021-08-04

**Authors:** Clayton J. Curtis, Andrei V. Astashkin, Jeanet Conradie, Abhik Ghosh, Elisa Tomat

**Affiliations:** † Department of Chemistry and Biochemistry, The University of Arizona, 1306 East University Blvd., Tucson, Arizona 85721, United States; ‡ Department of Chemistry, University of the Free State, P.O. Box 339, Bloemfontein 9300, Republic of South Africa; § Department of Chemistry, UiT − The Arctic University of Norway, N-9037 Tromsø, Norway

## Abstract

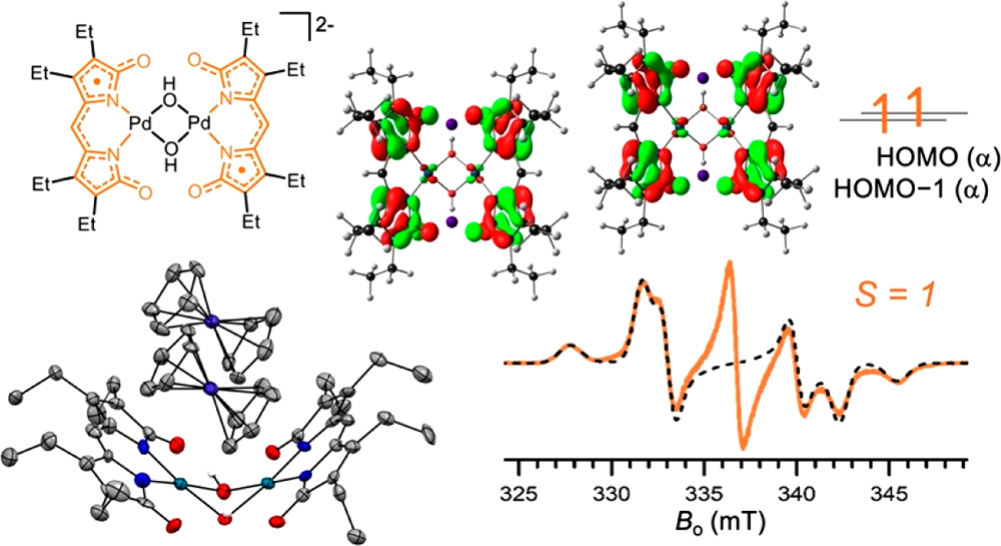

Oligopyrroles form a versatile class of redox-active ligands and electron reservoirs. Although the stabilization of radicals within oligopyrrolic π systems is more common for macrocyclic ligands, bidentate dipyrrindiones are emerging as compact platforms for one-electron redox chemistry in transition-metal complexes. We report the synthesis of a bis(aqua) palladium(II) dipyrrindione complex and its deprotonation-driven dimerization to form a hydroxo-bridged binuclear complex in the presence of water or triethylamine. Electrochemical, spectroelectrochemical, and computational analyses of the binuclear complex indicate the accessibility of two quasi-reversible ligand-centered reduction processes. The product of a two-electron chemical reduction by cobaltocene was isolated and characterized. In the solid state, this cobaltocenium salt features a folded dianionic complex that maintains the hydroxo bridges between the divalent palladium centers. X-band and Q-band EPR spectroscopic experiments and DFT computational analysis allow assignment of the dianionic species as a diradical with spin density almost entirely located on the two dipyrrindione ligands. As established from the EPR temperature dependence, the associated exchange coupling is weak and antiferromagnetic (*J* ≈ −2.5 K), which results in a predominantly triplet state at the temperatures at which the measurements have been performed.

## Abstract 2

The coordination and redox chemistry of the dipyrrindione scaffold, which is found in several heme metabolites, is investigated in heteroleptic palladium(II) complexes. The bis(aqua) complex undergoes a deprotonation-driven dimerization to form a hydroxo-bridged binuclear species. Crystallographic, electrochemical, and spectroscopic data, as well as computational analysis, demonstrate that a two-electron reduction of the binuclear complex leads to a diradical dianion with spin density delocalized over the two dipyrrindione ligands.

## Introduction

**Chart 1:**
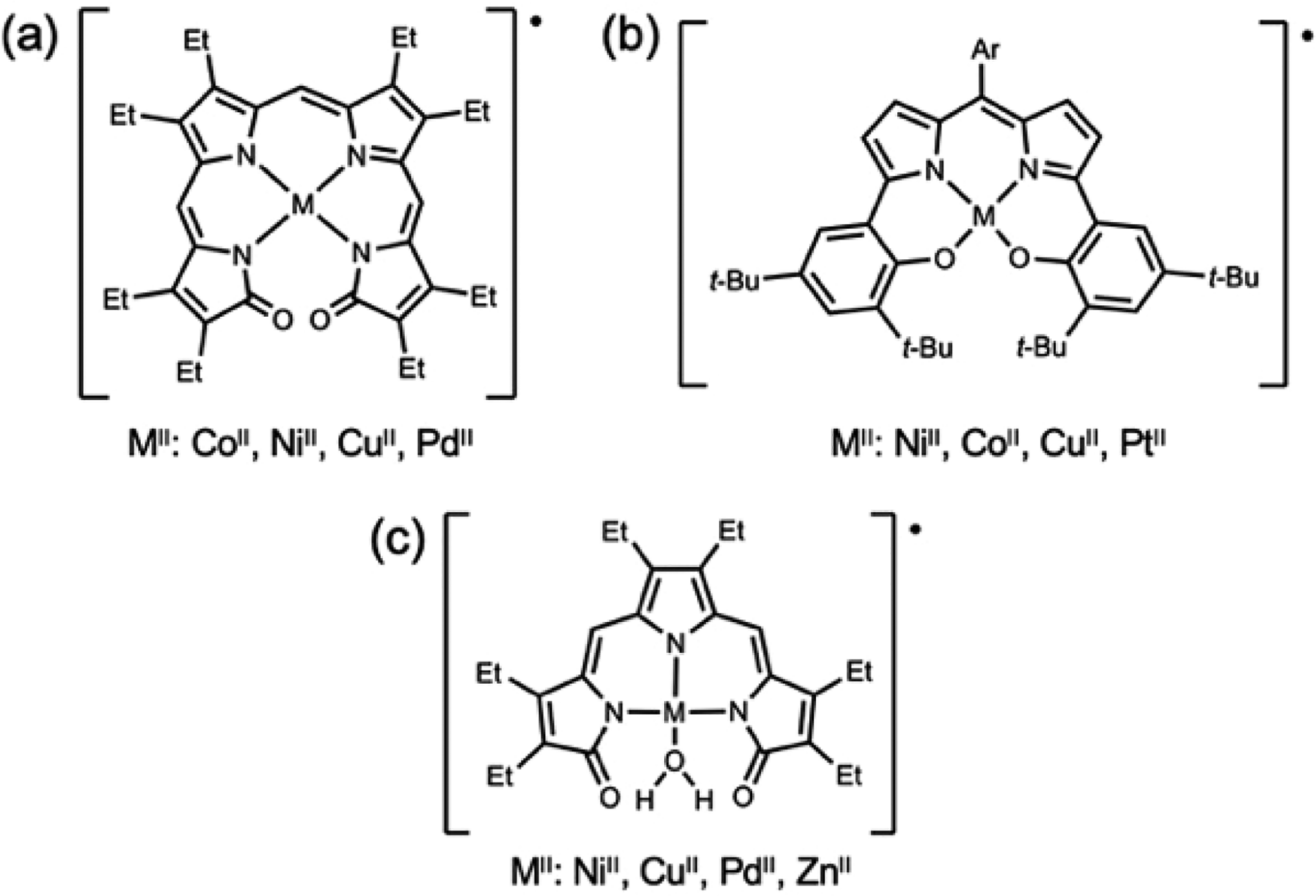
Metal Complexes of Tetradentate (a and b) and Tridentate (c) Pyrrolic Ligands Featuring Ligand-Based Radicals

Coordination compounds of redox-active ligands, in which oxidizing and/or reducing equivalents can be stored on the ligand π system, have generated broad interest owing to their potential applications in areas including homogeneous catalysis,^1^ molecular magnetism,^2^ photovoltaic devices,^3^ and quantum information processing (e.g., spin qubits).^4^ Macrocyclic oligopyrroles,^5^ such as porphyrins, corroles,^6^ and several expanded porphyrinoids,^7^ are well-studied examples of redox-active ligands, and their ability to host unpaired spins has been investigated extensively. Besides macrocyclic structures, early examples of linear oligopyrrolic radicals in metal complexes were found in the study of bilindiones (Chart 1a),^8,9^ but more recent reports highlighted the formation of stable ligand-based radicals in metal complexes of tetradentate bis(phenolate)-dipyrrins (Chart 1b),^10,11^ bis(2-aminophenyl)-dipyrrins,^12^ and diimino-dipyrrins,^13^ as well as tridentate tripyrrindione (Chart 1c)^14−18^ and dihydrazonopyrrole^19^ scaffolds.

Contracting the conjugated π system from tetradentate to tridentate to bidentate oligopyrroles, the observation of stable ligand-centered radicals becomes less common. Within the large family of bidentate dipyrrins,^20^ ligand-centered redox chemistry is observed with varying degrees of success depending on substituents;^21^ however, the bidentate dipyrrin system has not been generally employed for the stabilization of unpaired electrons. In contrast, the dipyrrin-1,9-dione motif serves as a compact dipyrrolic platform to host ligand-centered radicals.^22−24^ This bidentate ligand could therefore extend the diverse applications of dipyrrin complexes, ranging from fluorescent sensors to catalysis and metal–organic frameworks, to take advantage of one-electron redox chemistry and a variety of spin states.

**Chart 2:**
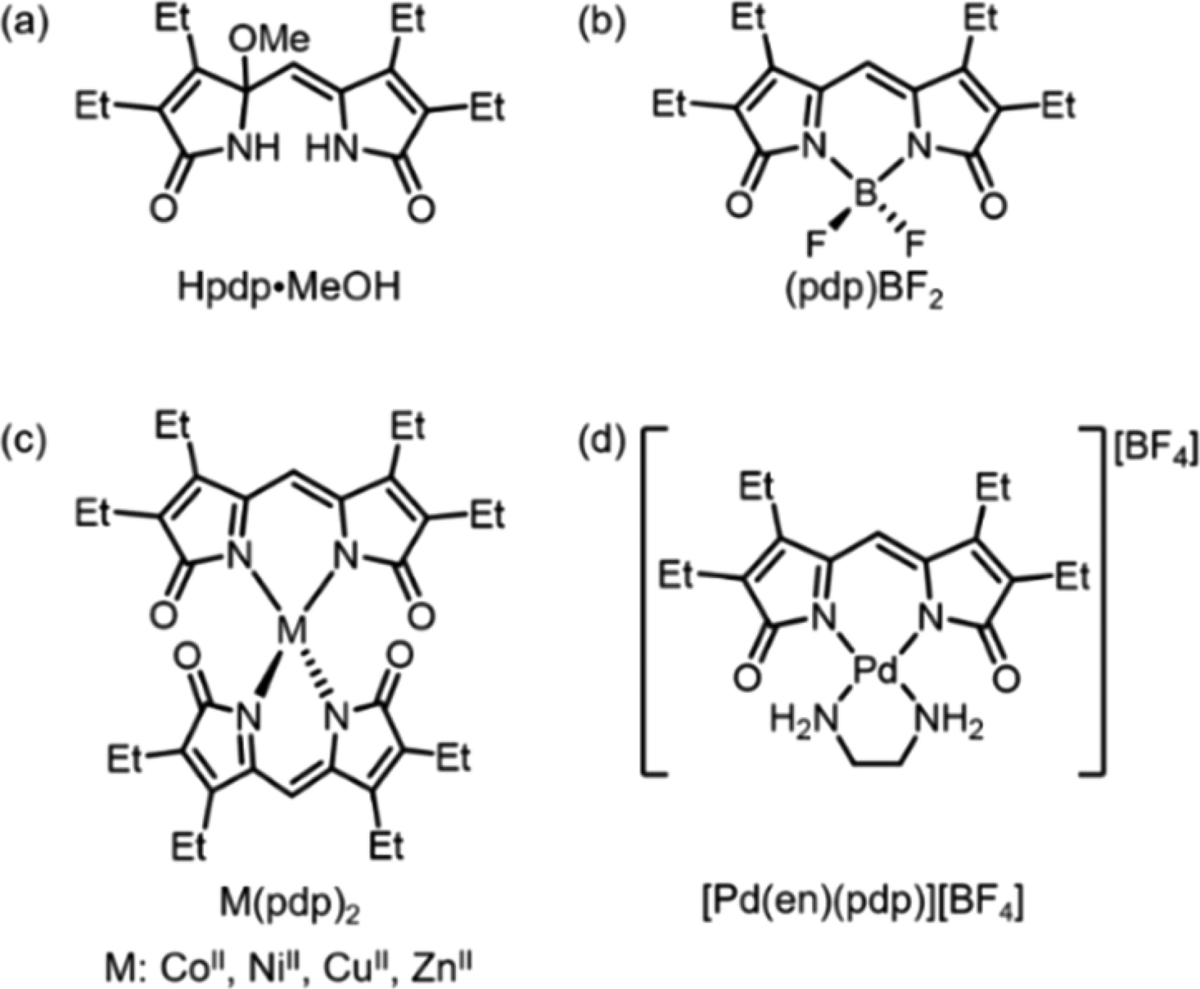
Tetraethyl Propentdyopent-Methanol Adduct (a) and Reported Complexes (b–d)

Historically referred to as propentdyopents, the naturally occurring dipyrrindione pigments result from the oxidative metabolism of heme.^25^ The first dipyrrindione complexes were isolated from products of degradation of bilindione complexes;^9,26^ however, we recently employed the Hpdp·MeOH adduct for the preparation of homoleptic^22^ and heteroleptic^23,24^ compounds (Chart 2). Because of the availability of low-lying π* molecular orbitals, these complexes typically undergo one-electron reduction processes localized on the dipyrrolic ligand framework. For instance, the fluorescence emission of BODIPY analogue (pdp)BF_2_ (Chart 2b) can be quenched through reduction of the dipyrrindione platform to form a ligand-based radical. In addition, spectroelectrochemical and electron paramagnetic resonance (EPR) measurements showed that two sequential one-electron reductions of Zn(pdp)_2_ (Chart 2c) lead to a (predominantly) triplet diradical complex, which was detected in solution.^22^

Diradical metal complexes featuring two ligand-based unpaired spins coordinated to diamagnetic metal centers (e.g., d^8^ or d^10^ ions) present a singlet or triplet ground state depending on the nature and relative orientation of the ligands.^27−29^ Reports on diradicals in bridged binuclear complexes featuring one redox-active ligand per metal center are rather scarce,^30,31^ but they point at an additional pathway for tuning spin exchange interactions between redox-active centers through modification of the bridging ligand(s). In this context, we sought to investigate the ability of the dipyrrindione framework to host unpaired spins in a bridged binuclear system. In our previous investigations of palladium(II) dipyrrindione complexes with primary amine ligands (e.g., [Pd(en)(pdp)]^+^, Chart 2d),^24^ we found that the intramolecular hydrogen bonds are important for complex stability. This observation prompted us to explore the synthesis of a similar diaqua complex. Given the acidity of palladium-bound aqua ligands,^32^ we reasoned that a diaqua complex would serve as a precursor to a bridged μ-hydroxo dimer, in turn allowing access to a dipyrrindione diradical species upon reduction.

## Results and Discussion

### Synthesis and Chemical Characterization

The tetraethyl dipyrrindione methanol adduct (Hpdp·MeOH, Chart 2a) was added to a dichloromethane solution of palladium(II) acetylacetonate in the presence of HBF_4_, which facilitated the dissociation of the bidentate acac^–^ ligands and their replacement with a higher-affinity ligand.^33^ This particular acid was also chosen so as to include the noncoordinating counterion BF_4_^–^ and facilitate crystallization. The reaction progress was accompanied by a color change from yellow to deep red over the course of 3–4 h at room temperature: in particular, we observed the gradual decrease of the single absorption band of Hpdp·MeOH at 280 nm and the growth of two main bands at 382 and 545 nm, consistent with our previous reports on dipyrrindione complexes.^22,24^

**Figure 1:**
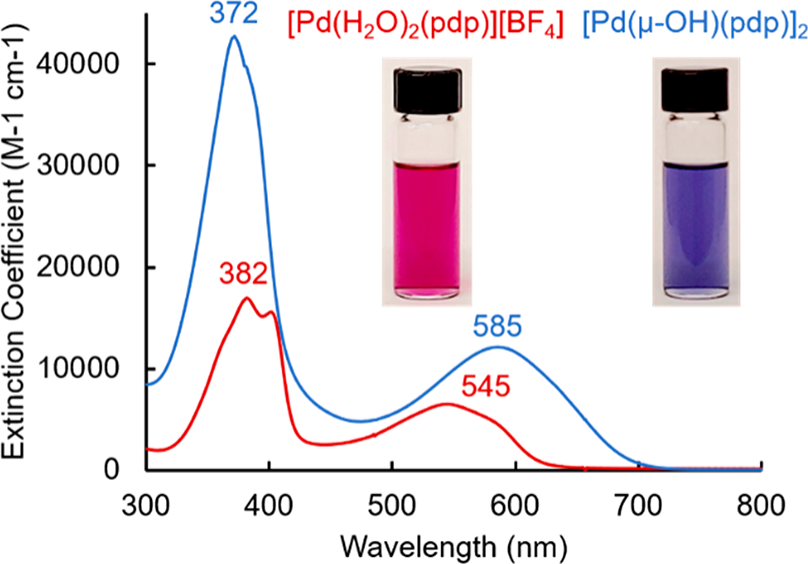
UV–visible absorption spectra of [Pd(H_2_O)_2_(pdp)][BF_4_] (red) and [Pd(μ-OH)(pdp)]_2_ (blue) in CH_2_Cl_2_.

In the course of our synthetic manipulations, we observed that exposing a dichloromethane solution of this red complex to an aqueous wash resulted in an immediate color change of the organic phase from red to blue. The absorption spectrum of the blue solution in CH_2_Cl_2_ presents a shift in the main bands to 372 and 585 nm (Figure 1 and Table S1), clearly indicating the formation of a different species.

The isolated red and blue complexes are diamagnetic, as determined by NMR spectroscopy. The ^1^H NMR spectrum of the red complex (Figure S1) features pdp^–^ resonances analogous to those in our previous reports.^24^ The corresponding ^13^C NMR spectrum (Figure S2) featured only resonances attributed to the dipyrrindione framework, eliminating the possibility of a bound acac^–^ ion. In addition, a sharp singlet integrating to four protons at 6.66 ppm in the ^1^H NMR spectrum suggested the presence of two coordinated aqua ligands bound to the metal center.

In the ^1^H and ^13^C NMR spectra of the blue complex (Figures S1 and S2), the resonances corresponding to the ligand framework were shifted slightly upfield relative to the red complex (e.g., 5.55 ppm vs 5.87 ppm for the *meso*-type proton). The most notable difference between the spectra was the disappearance of the 4H singlet at 6.66 ppm and the appearance of a new singlet at 3.07 ppm, which integrated to one proton relative to the *meso*-type resonance. Because of the increased acidity of palladium-bound aqua ligands, we hypothesized a deprotonation and formation of a hydroxo-bridged species as previously observed in the case of diaqua palladium(II)-BINAP complexes, which readily formed hydroxo dimers upon treatment with 4 Å molecular sieves in acetone.^34^ Conclusive data on the structure of both complexes in the solid state were then obtained by X-ray crystallography (see Table S2 for collection parameters).

**Figure 2:**
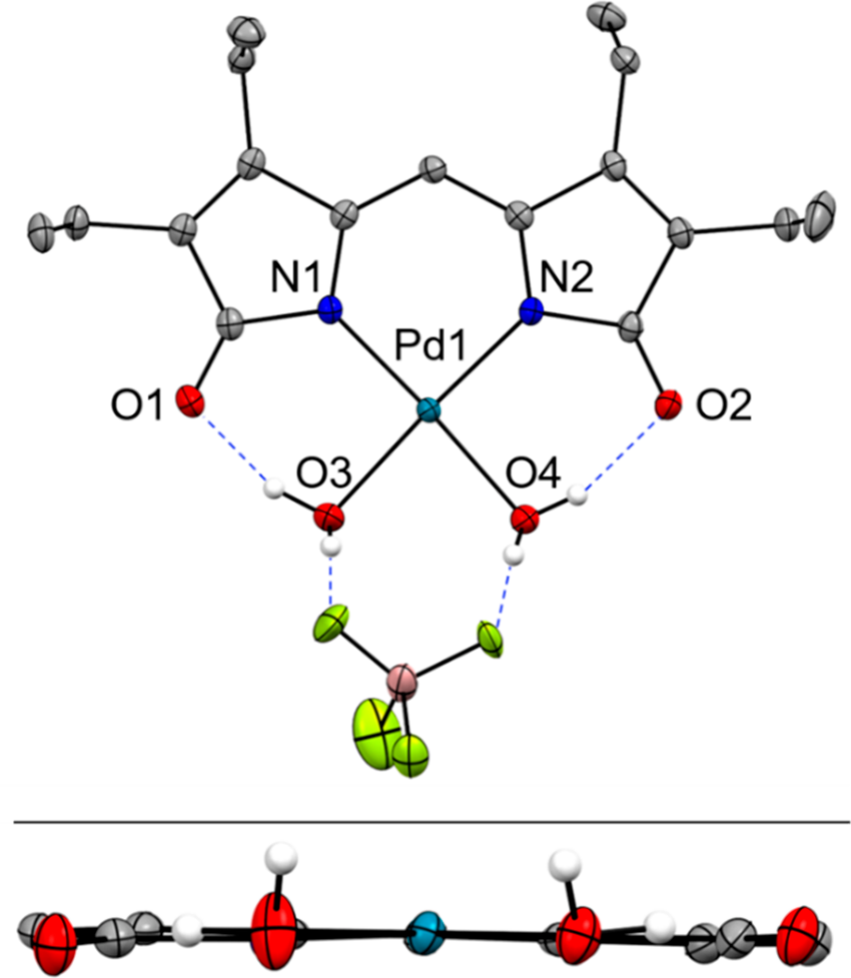
Crystal structure of [Pd(H_2_O)_2_(pdp)][BF_4_] showing a partial labeling scheme. Carbon-bound hydrogen atoms are omitted for clarity. Non-hydrogen atoms are displayed as thermal displacement ellipsoids set at the 50% probability level. In the side view (bottom panel), ethyl substituents were removed for clarity. CCDC: 2077676.

The crystal structure of the red complex confirmed the coordination of a single pdp^–^ ligand to the palladium(II) center also featuring two aqua ligands and a tetrafluoroborate counteranion in close vicinity (Figures 2 and S3). The coordination geometry of the palladium(II) center is square planar, and the Pd–N/O bond distances (ranging from 1.984(3) to 2.060(3) Å) and angles (84.3–92.6°) are consistent with those in analogous palladium(II) dipyrrinato complexes^35,36^ and heteroleptic dipyrrindione palladium(II) structures.^24^ The pdp^–^ ligand is fully coplanar with the palladium(II) coordination plane, and the bond lengths in the dipyrrindione framework (Table S3) indicate two terminal carbonyl groups at the pyrrolic α-positions (C–O, 1.223(3) and 1.226(3) Å). As previously reported for dipyrrindione^24^ and tripyrrindione^14,15,18^ complexes, these carbonyl groups serve as effective hydrogen-bonding acceptors: the aqua ligands are engaged in hydrogen-bonding interactions with both the propentdyopent scaffold and the tetrafluoroborate anions, and all aqua hydrogens were located on the Fourier map during structure refinement.

**Figure 3:**
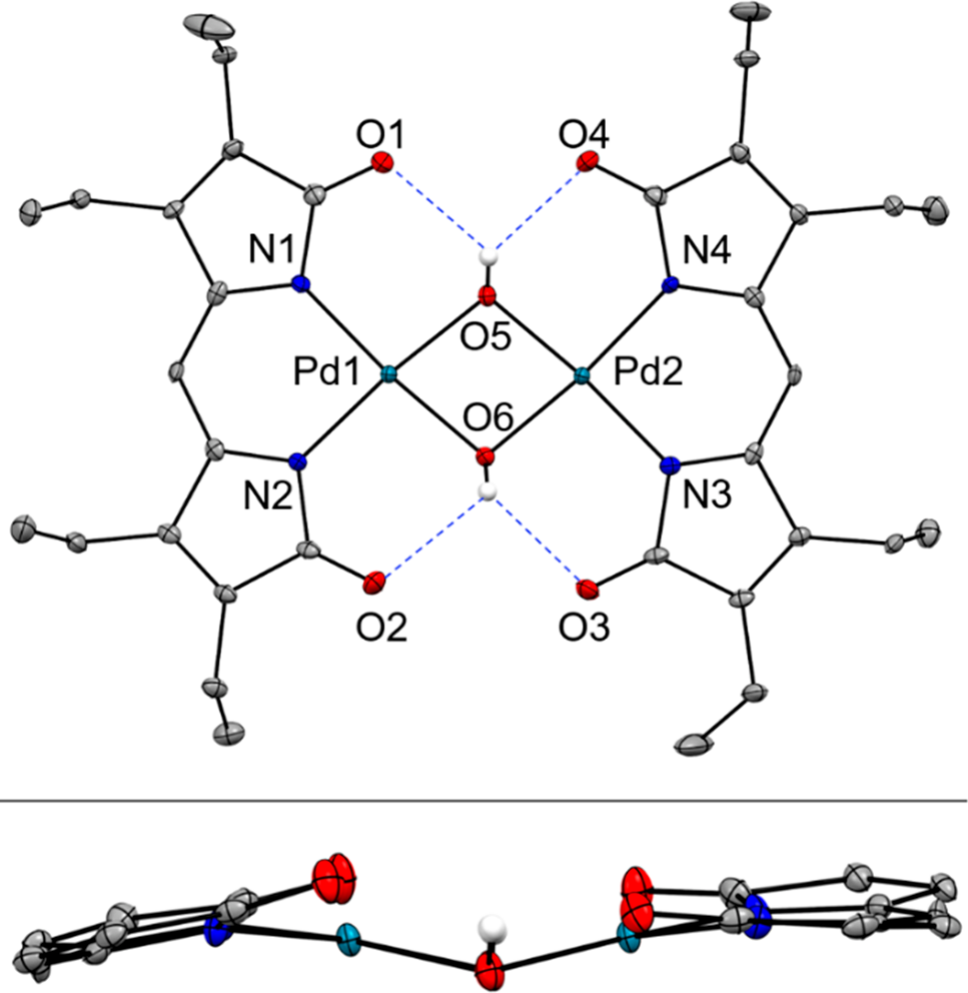
Crystal structure of [Pd(μ-OH)(pdp)]_2_ showing a partial labeling scheme. Carbon-bound hydrogen atoms are omitted for clarity. Non-hydrogen atoms are displayed as thermal displacement ellipsoids set at the 50% probability level. In the side view (bottom panel), ethyl substituents were removed for clarity. CCDC: 2077677.

The crystal structure of the blue complex (Figures 3 and S4) presents a coordination dimer, wherein the two palladium(II) centers are each bound to one dipyrrindione and two bridging hydroxo ligands. In contrast to the red complex [Pd(H_2_O)_2_(pdp)]^+^, the blue species [Pd(μ-OH)(pdp)]_2_ is neutral, and the two pdp^–^ ligands are slightly canted with a minor curvature of the rigid dipyrrolic scaffold. The Pd–N/O bond distances (1.979(3)–2.014(3) Å) are similar to those in [Pd(H_2_O)_2_(pdp)][BF_4_], and the bond lengths in the dipyrrolic framework remain largely unchanged upon dimerization (Table S4), indicating no change in the redox state of either the palladium(II) center or the ligand scaffold. The hydrogen atoms of the μ-hydroxo ligands are engaged in hydrogen-bonding interactions with two carbonyl oxygen atoms of the dipyrrindione ligands (Figure 3).

The solid-state analysis of [Pd(H_2_O)_2_(pdp)][BF_4_] and [Pd(μ-OH)(pdp)]_2_ is consistent with the observed differences between their respective ^1^H NMR spectra in CDCl_3_ solutions: the bound aqua ligands in the [Pd(H_2_O)_2_(pdp)]^+^ complex (i.e., 4H at 6.66 ppm) are deprotonated in aqueous conditions to form the dimeric complex [Pd(μ-OH)(pdp)]_2_ featuring two μ-hydroxo bridges (i.e., 2H at 3.07 ppm). The upfield shift of the OH resonance in the dimer is consistent with weaker hydrogen bonds, possibly owing to the less favorable orientation of the hydroxo bridges within the complex. For comparison, the aqua protons in the cationic Pd(II) tripyrrindione appear at 8.52 ppm, indicative of more effective hydrogen-bonding interactions.^14^

**Scheme 1:**
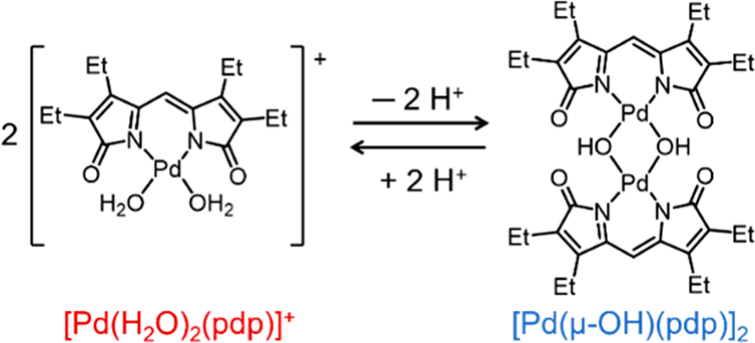
Reversible Deprotonation and Dimerization of [Pd(H_2_O)_2_(pdp)]^+^

The interconversion between [Pd(H_2_O)_2_(pdp)][BF_4_] and the *bis-*μ-hydroxo dimer [Pd(μ-OH)(pdp)]_2_ (Scheme 1) could be monitored by optical absorption spectroscopy in organic solvents. When starting with a solution of [Pd(H_2_O)_2_(pdp)][BF_4_] (75 μM, CH_2_Cl_2_), the addition of triethylamine resulted in the rapid consumption of the diaqua complex (λ_max_ = 545 nm) and formation of the μ-hydroxo dimer (λ_max_ = 585 nm), with saturation reached at ∼2.0 equiv of base (Figure S5). Conversely, the addition of trifluoroacetic acid (TFA, 2 equiv) to a solution of [Pd(μ-OH)(pdp)]_2_ (51 μM, 95:5 (v/v) CH_2_Cl_2_:CH_3_OH) resulted in the rapid shift of the lower-energy maximum from 585 to 545 nm, confirming the formation of the diaqua monomer complex (Figure S5). The addition of a small amount of methanol to the solvent mixture (i.e., 5% CH_3_OH in CH_2_Cl_2_) facilitated proton transfer in solution. Under these acidic conditions, however, partial demetalation was observed, particularly upon addition of more than 2.0 equiv of acid.

Overall, these experiments indicated the formation of a diaqua Pd(II) dipyrrindione complex that is similar to the analogous complexes of primary amines,^24^ in which the square planar coordination is stabilized by hydrogen-bonding interactions between ligands. The metal-bound aqua ligands, however, are quite acidic and undergo facile deprotonation concurrently with the formation of a *bis*(μ-OH) dimeric complex. A pronounced color change of the solution from red to blue accompanies the conversion of the diaqua complex to the *bis*-hydroxo species through deprotonation and dimerization.

### DFT Analysis of [Pd(H_2_O)_2_(pdp)]^+^ and [Pd(μ-OH)(pdp)]_2_

Density functional theory (DFT) calculations have been employed in one of our laboratories to describe ligand noninnocence,^37^ metal versus ligand-centered oxidation and reduction,^38,39^ and aromaticity^40,41^ in oligopyrrolic systems. Herein, all-electron dispersion-corrected scalar-relativistic DFT (OLYP-D3) calculations with large STO-TZ2P basis sets were deployed to contextualize the experimental results on [Pd(H_2_O)_2_(pdp)]^+^ and [Pd(μ-OH)(pdp)]_2_.

Although the optimized bond distances and angles were generally in good agreement with experimental values (Table S5), a folded conformation was found for the binuclear complex (Figure S6), at odds with an essentially planar geometry in the solid state (Figure 3). The Pd–Pd distance (2.685 Å) in the folded conformation was shorter than the sum of the van der Waals radii (3.26 Å), indicating a potential bonding interaction. The MOs for this *C*_2*v*_ geometry show filled bonding and antibonding orbitals involving the Pd(d_*z*^2^_) orbitals (Figure S7) typical of d^8^–d^8^ interactions,^30,42^ in which an overall weakly bonding character results from mixing with the metal s and p_*z*_ orbitals and is a potential driving force for this conformation. Although the occurrence of this folded structure in solution cannot be ruled out, packing effects in the crystalline solid are likely responsible for the rather planar structure observed in the solid state (Figure 3).

**Figure 4:**
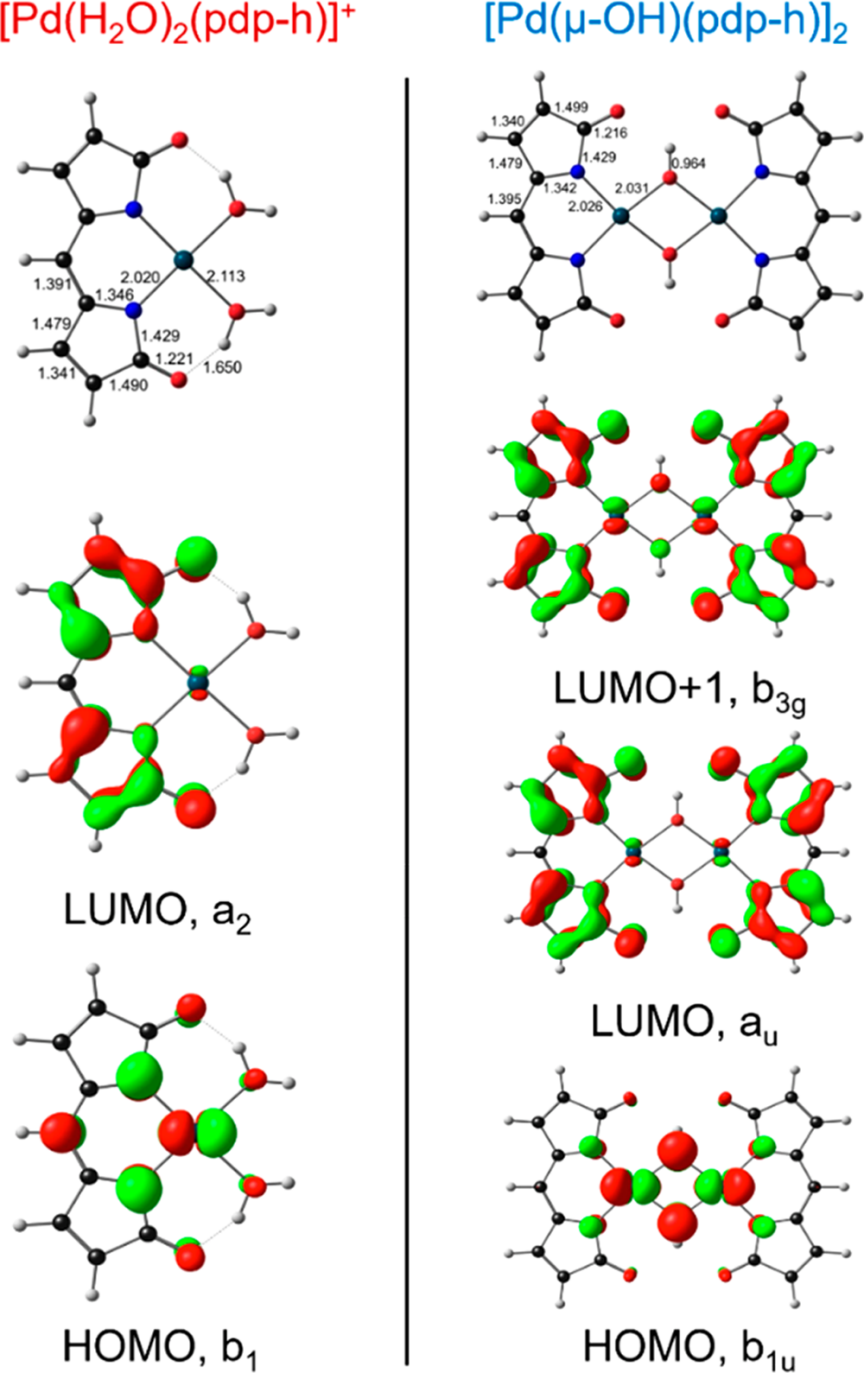
Frontier Kohn–Sham MOs of the simplified model complexes [Pd(H_2_O)_2_(pdp-h)]^+^ (*C*_2*v*_) and [Pd(μ-OH)(pdp-h)]_2_ (*D*_2*h*_) with selected bond lengths of optimized geometries (Å).

Because the folding of the dimer structure is likely to be soft mode with relatively little impact on intramolecular bonding, we chose to carry out further analysis with a symmetry-constrained *D*_2h_ (i.e., unfolded) model, [Pd(μ-OH)(pdp-h)]_2_, with unsubstituted pdp^–^ ligands. An examination of the HOMO and LUMO of the monomeric and dimeric complexes provided a plausible explanation for the 40 nm spectral redshift of the lowest-energy band in the UV–vis spectrum of the latter. Broadly speaking, the HOMOs of both the monomeric and dimeric complexes show substantial mixing of Pd(4d) and pdp(π)-based orbitals (Figures 4 and S8). The LUMOs, in contrast, are almost purely pdp-based. Compared to [Pd(H_2_O)_2_(pdp-h)]^+^, the HOMO of [Pd(μ-OH)(pdp-h)]_2_ exhibits increased destabilizing filled–filled Pd(d_π_)–O(p_*z*_) antibonding interactions, which translate to a lower HOMO–LUMO gap and thus to the observed spectral redshift.

### Characterization of Ligand-Based Reduction Products

**Figure 5:**
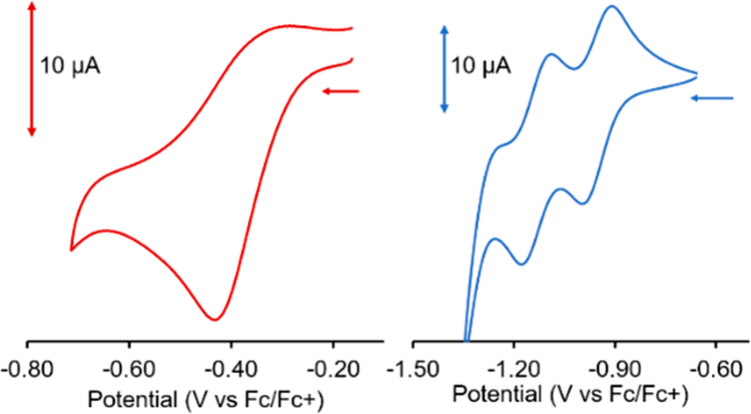
Cyclic voltammograms of [Pd(H_2_O)_2_(pdp)][BF_4_] (left) and [Pd(μ-OH)(pdp)]_2_ (right) at a glassy carbon electrode in CH_2_Cl_2_ with (NBu_4_)(PF_6_) as a supporting electrolyte. Data collected at a 100 mV s^–1^ scan rate using a Ag/AgCl pseudoreference electrode and a Pt wire auxiliary electrode.

The electrochemical profiles of [Pd(H_2_O)_2_(pdp)][BF_4_] and [Pd(μ-OH)(pdp)]_2_ were investigated by cyclic voltammetry in CH_2_Cl_2_, with potentials referenced to the ferrocenium/ferrocene redox couple (Fc^+^/Fc). The voltammogram of [Pd(H_2_O)_2_(pdp)][BF_4_] displayed an irreversible reduction event at −0.431 V (Figure 5, left panel). Spectroelectrochemical analysis of this event showed initial formation of absorption bands consistent with a dipyrrindione radical (Figure S9) as observed for other heteroleptic complexes.^24^ However, this species was short-lived, and a rapid overall decrease in absorbance intensity indicated degradation. In contrast, the cyclic voltammogram of [Pd(μ-OH)(pdp)]_2_ presented two quasi-reversible reduction events with half-wave potentials at −0.953 V (Δ*E*_p_ = 84 mV) and −1.134 V (Δ*E*_p_ = 86 mV) (Figure 5, right panel) (Figures S10 and S11). The cathodic shift of these potentials relative to that of the diaqua precursor (see above) is attributed to the cationic nature of the latter, which is more easily reduced.^24^ Overall, the electrochemical profile of [Pd(μ-OH)(pdp)]_2_ is consistent with two sequential ligand-centered reductions. The relatively small difference in the two half-wave potentials (181 mV) is suggestive of slight delocalization of the first reducing equivalent over the two ligands, corresponding to class II in the Robin–Day classification of mixed-valence compounds.^43^ Alternatively, assuming localized ligand reductions, the shift of the second reduction potential could be attributed to solvation effects of the monoanionic species as in the homoleptic propentdyopent complexes.^22^ In our theoretical model, we obtained a localized radical on one of the ligands for the singly reduced complex, as discussed below.

**Figure 6:**
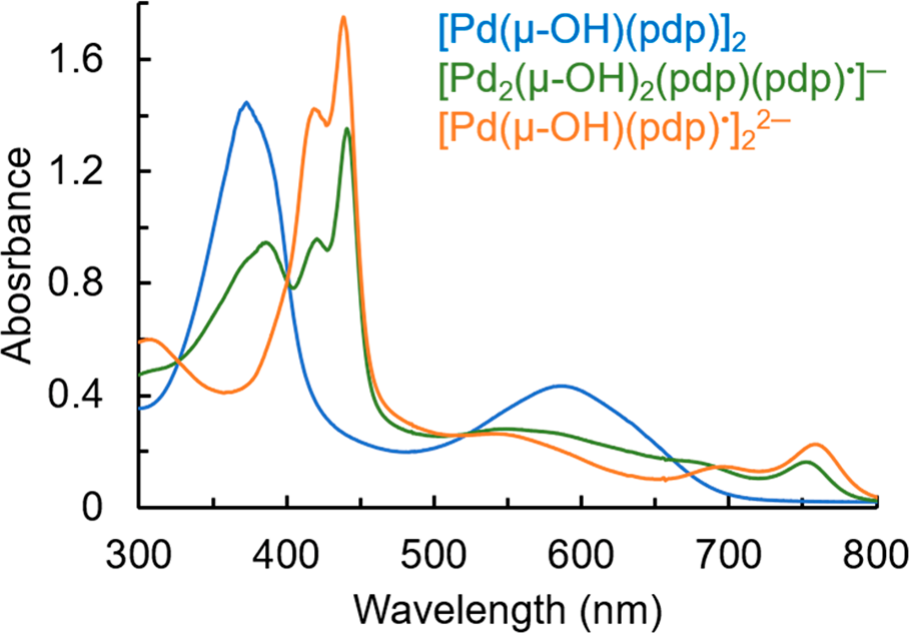
Spectral changes observed upon reduction of [Pd(μ-OH)(pdp)]_2_ (blue trace) (7:3 v/v DMF:CH_2_Cl_2_, 0.1 M (NBu_4_)(PF_6_)) by controlled potential electrolysis to produce the one-electron reduction product at −1.00 V (green trace) and then the two-electron reduction product at −1.30 V (orange trace).

The redox chemistry of [Pd(μ-OH)(pdp)]_2_ was further investigated by spectroelectrochemical methods (Figure 6). Upon controlled potential electrolysis at −1.00 V, the first reduction is accompanied by a decrease in the main absorption bands (372 and 585 nm) with concurrent appearance of transitions at 441, 675, and 752 nm. The presence of low-intensity, near-IR bands in the monoanionic complex is consistent with intraligand π–π charge-transfer transitions associated with oligopyrrolic radicals, as previously reported.^22−24,44^ Furthermore, the intensity of these bands increases upon generation of the doubly reduced species by electrolysis at −1.3 V, with minor wavelength shifts (to 439, 695, and 758 nm), suggesting the formation of a dianionic diradical.

The reduction of [Pd(μ-OH)(pdp)]_2_ by chemical methods was investigated using cobaltocene (CoCp_2_) in dichloromethane solutions. Anaerobic addition of either 1 or 2 equiv of CoCp_2_ at room temperature led to the rapid formation of green (1 equiv) or orange (2 equiv) solutions, with absorption spectra identical to those of the electrochemically generated products. Exposure of the reduced species to air resulted in reoxidation to the neutral complex [Pd(μ-OH)(pdp)]_2_ along with partial decomposition as monitored *via* absorption spectroscopy (Figure S12).

**Figure 7:**
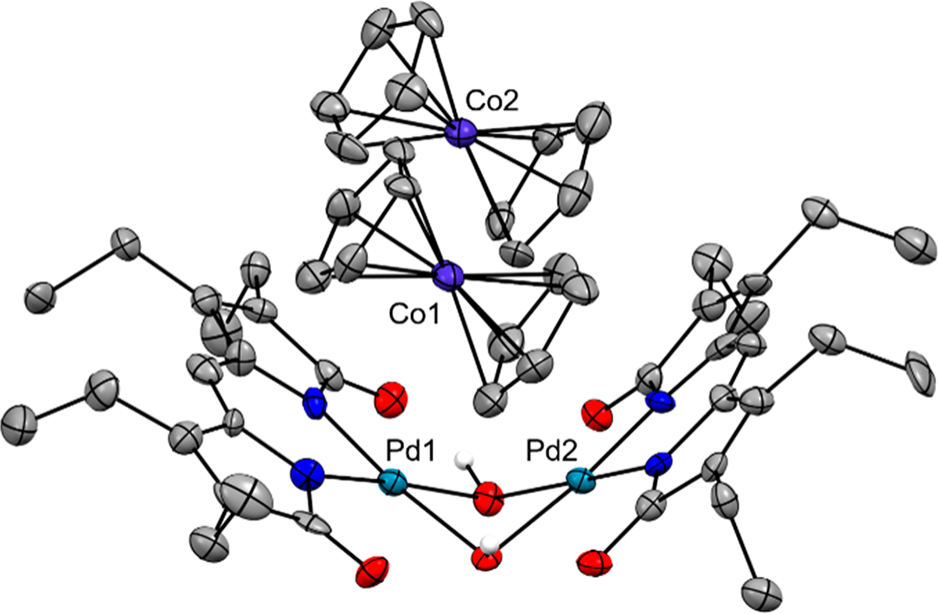
Crystal structure of [CoCp_2_]_2_[Pd(μ-OH)(pdp)^•^]_2_ showing a partial labeling scheme. Carbon-bound hydrogen atoms and solvent molecules are omitted for clarity. Non-hydrogen atoms are displayed as thermal displacement ellipsoids set at the 50% probability level. CCDC: 2077678.

The doubly reduced product was isolated from the reaction mixture by precipitation with pentane to afford a brown solid, and X-ray quality crystals were grown from a layered solution of CH_2_Cl_2_/pentane at −20 °C under a nitrogen atmosphere. The molecular structure obtained for these crystals (Figures 7 and S13) reveals the presence of two cobaltocenium counterions, thus confirming the successful formation of the two-electron reduction product. Readily apparent upon comparison to the neutral dimer is the increase in folding across the bridged hydroxo ligands: for instance, the angle between the two palladium(II) coordination planes contracts from 159° to 122° (Figure S14). The bonds between palladium centers and nitrogen/oxygen donors show slight elongation upon reduction but maintain the square planar geometry expected for a divalent oxidation state. More significant alterations are observed in the dipyrrindione ligand scaffold, particularly elongations of the terminal C–O and alternate contractions/elongations of C–N bonds on the pyrrolic rings (Table S4). Similar changes to the ligand framework were also observed in palladium(II) tripyrrindione complexes upon one-electron reduction,^14^ as well as in a bis(dipyrrinato)-Pacman dichromium complex after treatment with potassium graphite reductant.^45^ Overall, these changes in the dipyrrindione bond lengths support the assignment of two subsequent one-electron reductions to the ligand frameworks in [CoCp_2_]_2_[Pd(μ-OH)(pdp)^•^]_2_.

**Figure 8:**
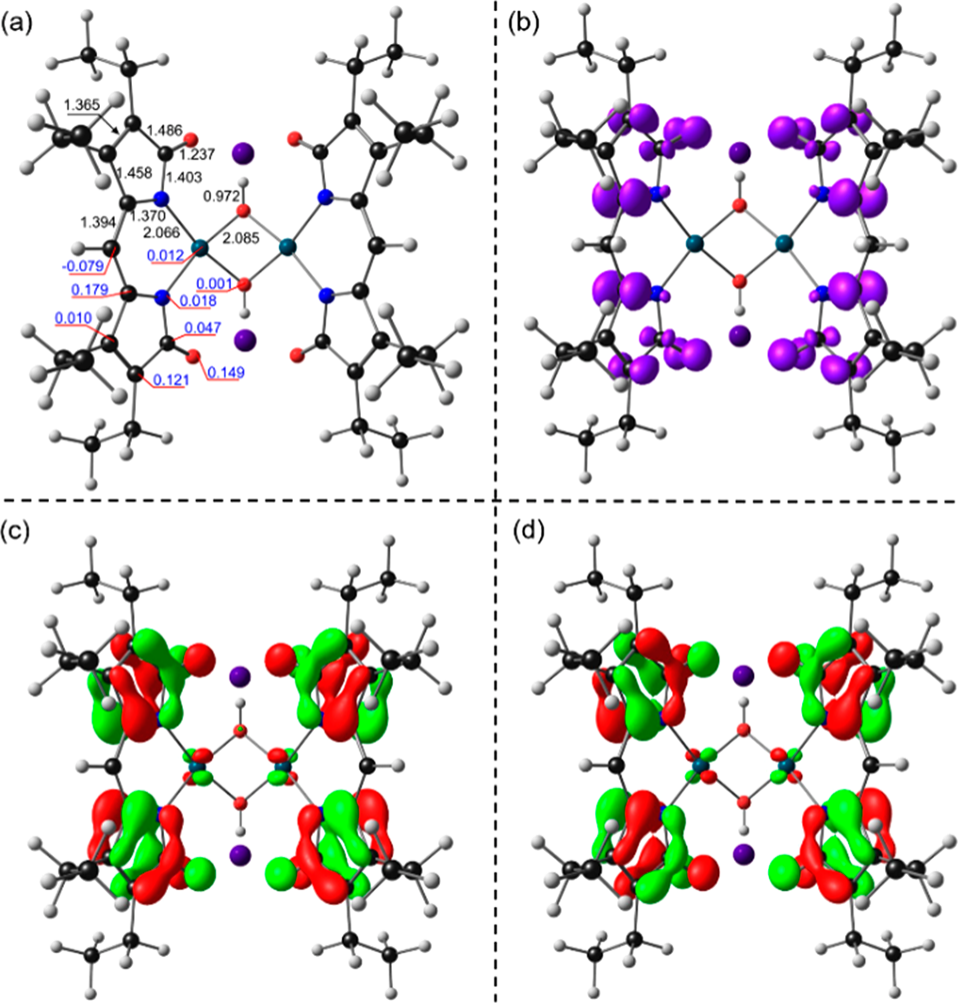
OLYP-D3/TZ2P computational results for diradical species Cs_2_[Pd(μ-OH)(pdp)^•^]_2_ showing (a) selected bond distances (Å) and Mulliken spin populations in black and blue, respectively, (b) spin densities in violet, and (c–d) SOMOs with phases indicated in red and green.

OLYP-D3/TZ2P calculations were deployed to simulate both monoanionic and dianionic reduction products *via* the model complexes Cs[Pd_2_(μ-OH)_2_(pdp)(pdp)^•^] and Cs_2_[Pd(μ-OH)(pdp)^•^]_2_, respectively. For the one-electron reduction product, the spin density was found to be localized on one of the dipyrrindione ligands (Figure S15), consistent with the spectroelectrochemical data showing the individual absorption profiles of the two ligands in two different redox states (Figure 6). For Cs_2_[Pd(μ-OH)(pdp)^•^]_2_, the bond lengths of the dipyrrindione scaffold were found to be in good agreement with the crystallographic data (Figures 8a and S16 and Table S6), whereas the calculated structure presents slightly contracted Pd–Pd distance (2.790 Å) and fold angle between dipyrrindione ligand planes (Table S8), likely associated with the size difference between the cobaltocenium and cesium cations. As expected for the product of two ligand-centered reductions, the Mulliken spin density is almost exclusively localized on the two dipyrrindione scaffolds (Figure 8b and Table S7). The two SOMOs (Figures 8 and S17) were found to be nearly degenerate and of predominantly ligand character, mirroring the spin density contours.

**Figure 9:**
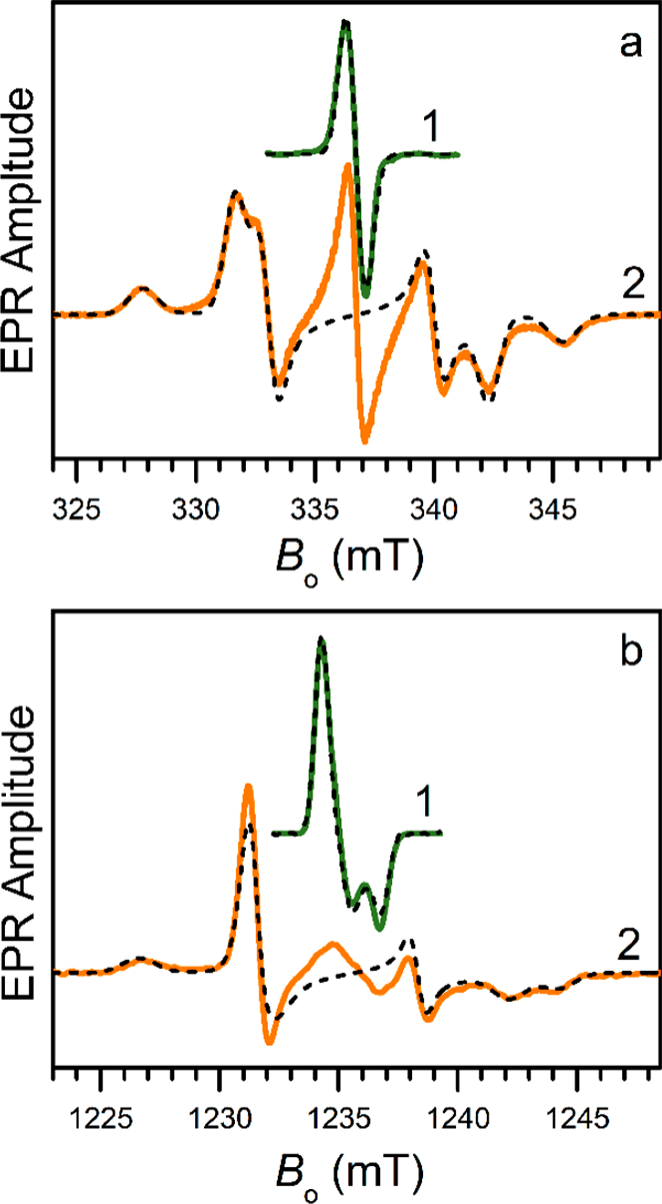
X-band (a) and Q-band (b) EPR spectra of the reduction products of [Pd(μ-OH)(pdp)]_2_. Traces 1 and 2 correspond to the reduction with 1 and 2 equiv of CoCp_2_, respectively. Simulated spectra are shown by black dashed lines. The simulation parameters are as follows: monoanionic complex g-tensor, (*g*_1_, *g*_2_, *g*_3_) = (2.0059, 2.0046, 2.0019); dianionic complex g-tensor, (*g*_*x*_, *g*_*y*_, *g*_*z*_) = (2.0045, 2.0047, 2.0022) and D-tensor, (*D*_*x*_, *D*_*y*_, *D*_*z*_) = (−330, 130, 200) MHz. Experimental conditions and individual line widths used in the simulations are detailed in the Experimental Section.

Further insights into the electronic structure of the reduction products were pursued by X-band and Q-band EPR measurements. The X-band EPR spectrum of the one-electron reduction product is dominated by a single Gaussian line with the width of 0.85 mT between the maximum slope points (Figure 9a, green trace 1). The zero crossing of this line at *B*_0_ = 336.7 mT corresponds to *g* ≈ 2.0038. The Q-band EPR measurement and spectrum simulation (Figure 9b, trace 1) resulted in a g-tensor with principal components (*g*_1_, *g*_2_, *g*_3_) = (2.0059, 2.0046, 2.0019). Such spectroscopic features are characteristic of an organic radical and confirm the ligand-based identity of the observed paramagnetic center. The X-band spectrum was successfully simulated (Figure 8a, dashed black trace 1) based on the g-tensor parameters found from Q-band data.

For a two-electron reduction product featuring two ligand-based spins, a strong magnetic dipole interaction between the unpaired electrons is expected, and indeed, the X-band EPR spectrum (Figure 9a, orange trace 2) shows the characteristic features of a rhombic dipolar coupling. Notably, the spectrum is not exactly symmetric with respect to the central line that corresponds to a residual singly reduced complex. As confirmed by the Q-band spectra (Figure 9b, orange trace 2), this asymmetry is caused by the g-factor anisotropy. The spectra were simulated successfully (Figure 9, black dashed traces) using a dipole interaction tensor (*D*_*x*_, *D*_*y*_, *D*_*z*_) = (−330, 130, 200) MHz aligned with the g-tensor (*g*_*x*_, *g*_*y*_, *g*_*z*_) = (2.0045, 2.0047, 2.0022). An analysis based on the spin density distributions predicted by DFT calculations shows (Table S7 and Figure S17) that the principal axis z coincides with the *C*_2_ symmetry axis of the dimeric complex (which has an overall *C*_2*v*_ symmetry); axis *x* points in the Pd-to-Pd direction, and axis *y* is parallel to the line joining the bridging oxygen atoms (Figure S19). This analysis also indicates that the angle between the ligand planes in the dianionic complex in solution is apparently close to 101.6° as observed in the crystal structure (Figure 7).

From the EPR temperature dependence at cryogenic temperatures (Figure S20), the exchange coupling between the unpaired electrons in the dianionic complex was estimated as *J* = −1.75 ± 0.35 cm^–1^ (−2.5 ± 0.5 K), thus indicating a weak antiferromagnetic exchange interaction. Although the ground state is singlet, the *J*-value is small, and at high temperatures (*kT* ≫ *J*), about 75% of the complexes are in the triplet state.

As an additional corroboration of the triplet state assignment, we have observed the half-field *ΔM* = 2 EPR transition for the dianionic complex (see Figure S21). Collectively, the EPR data have fully confirmed the assignment of [Pd(μ-OH)(pdp)^•^]_2_^2–^ as a diradical delocalized on the dipyrrindione ligands.

## Conclusions

The coordination and redox chemistry of tetraethyl dipyrrindione, also known as propentdyopent, was investigated in heteroleptic palladium complexes. The bis(aqua) Pd(II) dipyrrindione complex readily undergoes a deprotonation-driven dimerization in the presence of water to give a hydroxo-bridged binuclear complex. The dimerization is accompanied by a 40 nm redshift in the visible absorption spectrum and is reversible upon addition of stoichiometric amounts of protic acid. The binuclear μ-hydroxo complex maintains the reversible ligand-centered redox chemistry of prior mononuclear dipyrrindione complexes and undergoes two quasi-reversible one-electron reduction events. Chemical reduction of this binuclear complex by cobaltocene led to the doubly reduced product. A detailed analysis by crystallography, EPR spectroscopy, and DFT calculations confirmed the formation of a dianionic diradical species, wherein two unpaired electrons are coupled by a weak antiferromagnetic exchange interaction. The weak exchange coupling (*J* ≈ −2.5 K) results in a predominantly triplet spin state at our usual measurement temperatures of 77 or 296 K (room temperature). In addition, the dipolar interaction parameters obtained by EPR experiments are consistent with the folded structure observed in the solid state, possibly featuring a d^8^–d^8^ interaction between the metal centers. The effects of different metal ions and bridging ligands on the geometry and spin interactions of binuclear dipyrrindione complexes will be the subject of upcoming studies. This work demonstrates conclusively the stabilization of unpaired spins on the compact bidentate scaffold of dipyrrindione ligands, thus enhancing the scope of the large family of dipyrrins widely employed in catalysis, imaging, and materials science.

## Experimental Section

### Materials and Methods

The tetraethyl dipyrrin-1,9-dione methanol adduct (Hpdp·MeOH)^46^ and palladium(II) acetylacetonate (Pd(acac)_2_)^47^ were prepared as previously reported. Dichloromethane (CH_2_Cl_2_), dimethylformamide (DMF), and pentane were dried by passage through a solvent purifier. All other commercial reagents were used without further purification. NMR spectra were recorded on a Bruker Advance-III 400 MHz and a Bruker NEO-500 MHz NMR spectrometer at the NMR Spectroscopy Facility of the Department of Chemistry and Biochemistry. UV–visible spectra were obtained at ambient temperature using an Agilent 8453 UV–vis spectrophotometer. High-resolution mass spectra (HRMS) *via* electrospray ionization (ESI) methods were obtained at the University of Arizona Analytical & Biological Mass Spectrometry Core Facility. Elemental analyses were performed by NuMega Resonance Laboratories in San Diego, CA.

### Synthetic Procedures

#### [Pd(H_2_O)_2_(pdp)][BF_4_]

Hpdp·MeOH (8.5 mg, 0.027 mmol) and Pd(acac)_2_ (8.1 mg, 0.027 mmol) were dissolved in CH_2_Cl_2_ (10 mL), and HBF_4_ (10 μL, 48% aqueous solution) was added to the reaction mixture; the mixture was stirred at room temperature for 3 h. Upon reaction completion, as determined through UV–visible absorption spectroscopy, the solvent was removed *in vacuo*, and the red solid was purified by crystallization from a layered solution of CH_2_Cl_2_ and pentane to afford [Pd(H_2_O)_2_(pdp)][BF_4_] as a red crystalline solid (6.0 mg, 43%). ^1^H NMR (500 MHz, CDCl_3_): δ 6.66 (s, 4H), 5.87 (s, 1H) 2.54 (q, *J* = 7.7 Hz, 4H), 2.35 (q, *J* = 7.6 Hz, 4H), 1.21 (t, *J* = 7.7 Hz, 6H), 1.13 (t, *J* = 7.6 Hz, 6H). ^13^C NMR (125 MHz, CDCl_3_): δ 185.29, 168.78, 149.90, 141.85, 94.92, 17.87, 17.59, 14.27, 13.16. UV–vis (CH_2_Cl_2_) λ_max_ (ε): 382 (17,400), 402 (16,000), 545 (6,500 M^–1^ cm^–1^). HRMS-ESI^+^ (*m*/*z*): [M]^+^ calcd. for [C_17_H_25_N_2_O_4_Pd], 427.0849; found, 427.0845. Anal. Calcd for [C_17_H_25_N_2_O_4_BF_4_Pd]: C, 39.7; H, 4.9; N, 5.4%. Found: C, 39.8; H, 4.5; N, 5.5%.

#### [Pd(μ-OH)(pdp)]_2_

[Pd(H_2_O)_2_(pdp)][BF_4_] (7.6 mg, 0.0148 mmol) was dissolved in CH_2_Cl_2_ (15 mL) and shaken with deionized water (20 mL) in a separatory funnel until the organic layer fully converted from red to blue. The organic layer was further washed with brine and dried over Na_2_SO_4_, and the solvent was removed *in vacuo*. The resulting blue solid was purified by crystallization from a layered solution of CH_2_Cl_2_ and pentane to afford [Pd(μ-OH)(pdp)]_2_ as a blue crystalline solid (5.3 mg, 88%). ^1^H NMR (500 MHz, CDCl_3_): δ 5.55 (s, 2H), 3.07 (s, 2H), 2.47 (q, *J* = 7.7 Hz, 8H), 2.33 (q, *J* = 7.6 Hz, 8H), 1.14 (t, *J* = 7.7 Hz, 12H), 1.07 (t, *J* = 7.6 Hz, 12H). ^13^C NMR (125 MHz, CDCl_3_): δ 182.04, 167.82, 148.04, 139.85, 91.66, 17.85, 17.39, 14.69, 13.70. UV–vis (CH_2_Cl_2_) λ_max_ (ε): 372 (42,600), 585 (12,400 M^–1^ cm^–1^). HRMS-ESI^+^ (*m*/*z*): [M + Na]^+^ calcd. for [C_34_H_44_N_4_O_6_Pd_2_Na], 841.1227; found, 841.1237. Anal. Calcd Ffor [C_34_H_44_N_4_O_6_Pd_2_]: C, 49.9; H, 5.4; N, 6.8%. Found: C, 49.8; H, 5.1; N, 6.9%.

#### [CoCp_2_]_2_[Pd(μ-OH)(pdp)^•^]_2_

[Pd(μ-OH)(pdp)]_2_ (7.8 mg, 0.0095 mmol) was dissolved in CH_2_Cl_2_ (1.5 mL) in a glovebox. CoCp_2_ (5.2 mg, 0.0275 mmol) reductant was dissolved in CH_2_Cl_2_ (2.0 mL) to give a 13.75 mM stock solution, 1.40 mL (0.01925 mmol, 2 equiv) of which were added to the solution of [Pd(μ-OH)(pdp)]_2_. After 3 min of stirring, pentane was added to the reaction mixture to form a brown precipitate. The brown solid was isolated *via* vacuum filtration and washed with additional pentane to afford [CoCp_2_]_2_[Pd(μ-OH)(pdp)^•^]_2_ (8.0 mg, 70%). Anal. Calcd for [C_54_H_63_N_4_O_6_Pd_2_Co_2_·1.5CH_2_Cl_2_]: C, 50.4; H, 5.1; N, 4.2%. Found: C, 50.5; H, 4.9; N, 4.2%.

### X-ray Diffraction Analysis

The single crystal X-ray diffraction measurements were performed at the XRD facility of the University of Arizona, Department of Chemistry and Biochemistry, on a Bruker Kappa APEX II Duo diffractometer equipped APEX II CCD area detector, four-circle kappa goniometer, and an Oxford Cryostream low-temperature system. The data collection was performed at 100 K, using the Mo Kα radiation (λ = 0.71073 Å). During the measurements, the instrument was controlled by the *APEX2* software package (Bruker AXS Inc., Madison, WI, 2007). The absorption correction was done using a multiscan method implemented in *SADABS* (Sheldrick, G. M.; University of Göttingen, Germany, 1997).

The crystal structures were solved and refined using the *SHELX* package^48^ called from the *Olex2*(49) GUI. All non-H atoms were located in the Fourier map and were refined anisotropically. The carbon-bound hydrogen atoms were calculated in ideal positions with isotropic displacement parameters set to 1.2*U*_eq_ of the attached atom (1.5*U*_eq_ for methyl hydrogen atoms); their positions were then refined using a riding model. The relevant experimental and structure refinement details are available in Table S2.

#### Structure Refinement of [Pd(H_2_O)_2_(pdp)][BF_4_]

Crystals grew as red plates by slow diffusion of hexanes into a solution of CH_2_Cl_2_ at room temperature. Data were solved and refined in the triclinic space group *P*-1. The asymmetric unit cell contained one complex. *Q*-peaks for the hydrogen-bound O–H protons were located in the Fourier map, and hydrogens were assigned to those positions and refined. The four fluorine atoms (F1, F2, F3, and F4) of the tetrafluoroborate anion were found to be disordered by a rotational distribution around the boron atom. The disorder was modeled by a distribution over two positions to provide a stable refinement. The highest residual Fourier peak found in the model was +1.70 *e* Å^–3^ approximately 0.88 Å from F3A, and the deepest Fourier hole was −0.86 *e* Å^–3^ approximately 0.75 Å from F4.

#### Structure Refinement of [Pd(μ-OH)(pdp)]_2_

Crystals grew as red plates by slow diffusion of hexanes into CH_2_Cl_2_ at room temperature. Data were solved and refined in the triclinic space group *P*-1. The asymmetric unit cell contained three complexes. *Q*-peaks for hydrogen-bound O–H protons were located in the Fourier map, and hydrogens were assigned to those positions and refined. The highest residual Fourier peak found in the model was +0.98 *e* Å^–3^ approximately 0.89 Å from Pd1C, and the deepest Fourier hole was −0.58 *e* Å^–3^ approximately 0.74 Å from Pd2C.

#### Structure Refinement of [CoCp_2_]_2_[Pd(μ-OH)(pdp)^•^]_2_

Crystals grew as brown-orange plates by slow diffusion of pentane into CH_2_Cl_2_ at −20 °C. Data were solved and refined in the orthorhombic space group *P*2_1_2_1_2_1_. The highest residual Fourier peak found in the model was +1.05 *e* Å^–3^ approximately 1.29 Å from O6, and the deepest Fourier hole was −0.84 *e* Å^–3^ approximately 0.87 Å from Co2. The hydrogen atoms on bridging oxygens O5 and O6 were restrained to bond lengths of 1.00 Å using DFIX.

### Computational Methods

DFT calculations with full geometry optimizations were carried out with the ADF 2018 program system.^50^ A variety of exchange–correlation functionals were tested; the results quoted are those for OLYP,^51,52^ one of the better generalized gradient approximations that we have tested extensively on a variety of transition-metal-containing systems,^53−55^ augmented with Grimme’s D3^56^ dispersion corrections. All-electron Slater-type triple-z plus double polarization (STO-TZ2P) basis sets were used throughout. Point group symmetry was exploited, both in the interest of greater insight and to calculate electronic states with different orbital occupancies. Key results were checked against other pure, hybrid, and range-separated functionals and found to be stable.

### Electrochemical Measurements

Cyclic voltammograms were performed on a Gamry Reference 600 potentiostat utilizing a single-compartment cell with three electrodes: a glassy carbon working electrode, a platinum wire auxiliary electrode, and a Ag/AgCl quasi-reference electrode. Measurements were performed at ambient temperature under an inert argon atmosphere in CH_2_Cl_2_ containing 0.1 M (NBu_4_)(PF_6_) (triply recrystallized) as a supporting electrolyte. Sample concentrations were 1–2 mM, and all electrochemical data were internally referenced to the ferrocene/ferrocenium couple (set at 0.00 V). Spectroelectrochemical measurements were performed using a three-electrode electrochemical quartz cell with a 1.0 mm path length, a Au gauze working electrode, Ag wire quasi-reference electrode, and a Pt wire counter electrode. The complex was dissolved in 70:30 (v/v) DMF:CH_2_Cl_2_ containing 0.1 M (NBu_4_)(PF_6_).

### EPR Measurements and Simulations

The EPR measurements were performed at two microwave (mw) bands: X (∼9.5 GHz) and Q (∼34 GHz). In the X-band experiments, a continuous-wave EPR spectrometer Elexsys E500 (Bruker Biospin) equipped with a rectangular TE_102_ resonator was used with experimental conditions as follows: mw frequency, 9.441 GHz; mw power, 20 μW; magnetic field modulation amplitude, 0.2 mT. The measurement temperature of 77 K was achieved by using liquid nitrogen in a finger dewar.

The Q-band measurements were performed on a home-built pulsed EPR spectrometer^57^ equipped with a cylindrical TE_011_ resonator and a helium gas flow cryostat (CF935, Oxford Instruments). The EPR spectra were obtained by taking a numerical first derivative of electron spin echo (ESE) field sweep spectra. Experimental conditions: mw frequency, 34.644 GHz; mw pulse durations, 140 and 250 ns; time interval τ between the pulses, 350 ns; boxcar integration gate, 150 ns; measurement temperature, 15 K.

The numerical simulations of the EPR spectra were performed using custom software based on the exact diagonalization of the spin Hamiltonian. In the simulations of the spectrum of the dianionic complex, the *C*_2*v*_ symmetry was taken into account. This symmetry determines the directions of the magnetic axes with respect to the complex (along the *C*_2_ axis, along the line joining the bridging oxygen atoms, and along the line joining the Pd ions) and requires that the g- and D-tensors are coaxial. The spectroscopic parameters resulting from the simulations are as follows. For the one-electron reduction product, the g-tensor is (*g*_1_, *g*_2_, *g*_3_) = (2.0059, 2.0046, 2.0019). The individual line widths used are (*δB*_1_, *δB*_2_, *δB*_3_) = (0.75, 0.75, 0.75) mT for the X-band simulation and (*δB*_1_, *δB*_2_, *δB*_3_) = (0.5, 0.8, 0.65) mT for the Q-band simulation. The simulation parameters for the 2-electron reduction product are g-tensor, (*g*_*x*_, *g*_*y*_, *g*_*z*_) = (2.0045, 2.0047, 2.0022); D-tensor, (*D*_*x*_, *D*_*y*_, *D*_*z*_) = (−330, 130, 200) MHz; individual line widths for X-band, (*δB*_*x*_, *δB*_*y*_, *δB*_*z*_) = (1.27, 0.7, 0.85) mT; individual line widths for Q-band, (*δB*_*x*_, *δB*_*y*_, *δB*_*z*_) = (1.4, 0.6, 1.4) mT.

## Supporting Information Available

Optical absorption data, NMR data, X-ray crystallography data, electrochemical data, computational details, EPR analysis data, and supporting figures and tables (PDF)

## Accession Codes

CCDC 2077676–2077678 contain the supplementary crystallographic data for this paper. These data can be obtained free of charge via www.ccdc.cam.ac.uk/data_request/cif, or by emailing data_request@ccdc.cam.ac.uk, or by contacting The Cambridge Crystallographic Data Centre, 12 Union Road, Cambridge CB2 1EZ, UK; fax: +44 1223 336033.
