# Supplementary material for: Ligand-Centered Triplet Diradical Supported by a Binuclear Palladium(II) Dipyrrindione
Source: Inorg Chem. 2021 Aug 4;60(16):12457–66. doi: 10.1021/acs.inorgchem.1c01691 (PMC8389801; doi:10.1021/acs.inorgchem.1c01691)
Supplement: Supplementary file 1 — ic1c01691_si_001.pdf [file ic1c01691_si_001.pdf]

# Supporting Information

# Ligand-centered triplet diradical supported by a binuclear palladium(II) dipyrrindione

**Clayton J. Curtis,[a] Andrei V. Astashkin,[a] Jeanet Conradie,[b,c] Abhik Ghosh,[c*] and Elisa Tomat[a*]**

[a] Department of Chemistry and Biochemistry, The University of Arizona, 1306 E. University Blvd., Tucson AZ 85721, USA

[b] Department of Chemistry, University of the Free State, P.O. Box 339, Bloemfontein 9300, Republic of South Africa.

[c] Department of Chemistry, UiT – The Arctic University of Norway, N-9037 Tromsø, Norway

**Contents**

Optical absorption data (Table S1) S2

NMR data (Figs. S1 – S2) S2

X-ray diffraction analysis of monomer and dimer complexes (Figs. S3 – S4, Tables S2 – S4) S4

UV-vis absorption titrations (Fig. S5) S8

DFT analysis of monomer and dimer complexes (Figs. S6 – S8, Table S5) S9

Electrochemical data (Figs. S9 – S11) S11

Air oxidation of dianionic diradical (Fig. S12) S12

X-ray diffraction analysis of dianionic diradical (Figs. S13 – S14) S13

DFT analysis (Figs. S15 – S17, Tables S6 – S7) S14

EPR analysis (Figs. S18 – S21, Table S8) S16

Optimized cartesian coordinates S19

**Table S1.** Molar extinction coefficients of [Pd($H_2O$)$_2$(pdp)][$BF_4$] and [Pd(μ-OH)(pdp)]$_2$ complexes obtained in $CH_2Cl_2$.

| Complex | $\lambda_{max}$ (ε) / nm ($M^{-1}$ $cm^{-1}$) |
|---|---|
| [Pd($H_2O$)$_2$(pdp)][$BF_4$] | 382 (17,400), 402 (16,000), 545 (6,500) |
| [Pd(μ-OH)(pdp)]$_2$ | 372 (42,600), 585 (12,400) |

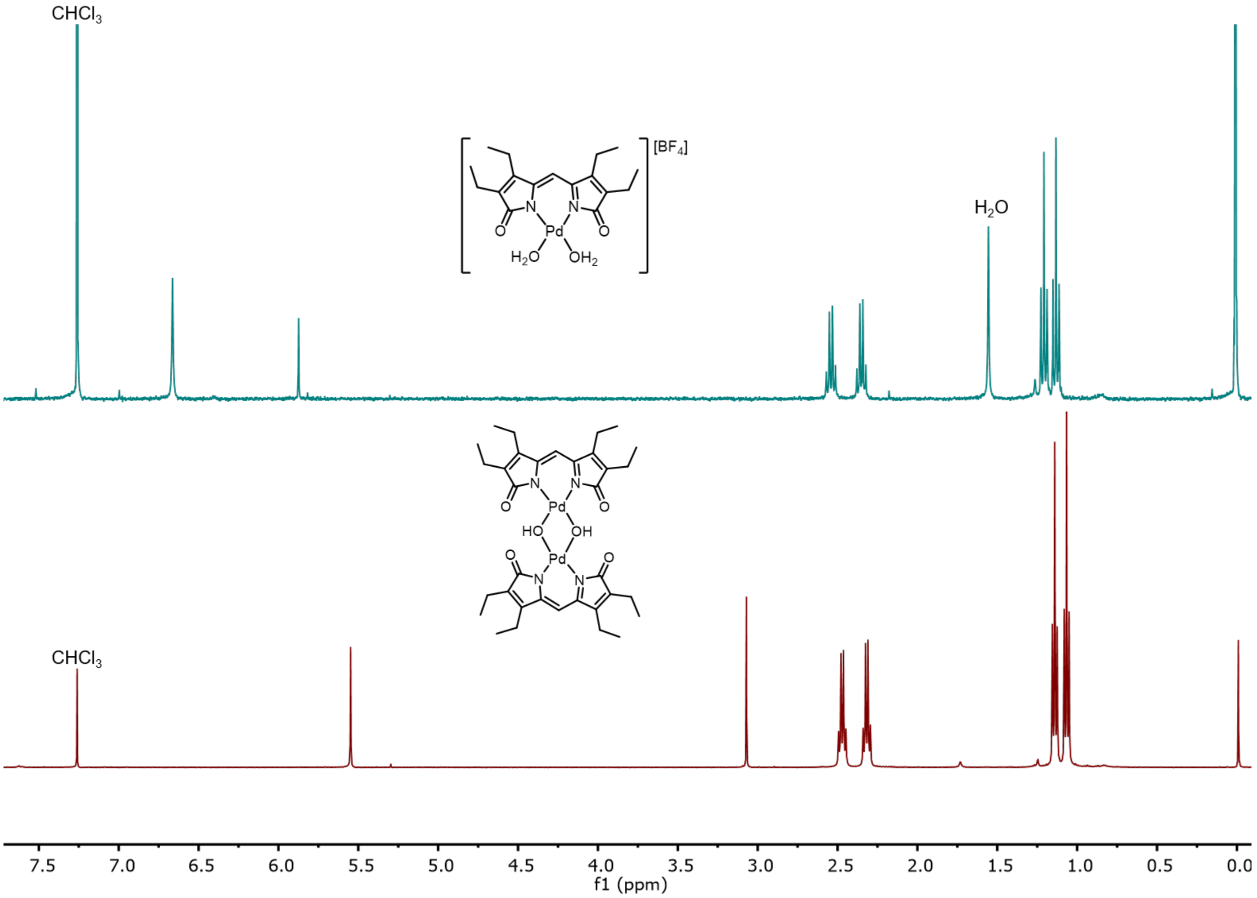


**Figure S1.** $^1$H NMR spectra of [Pd($H_2O$)$_2$(pdp)][$BF_4$] and [Pd(μ-OH)(pdp)]$_2$ complexes in $CDCl_3$.

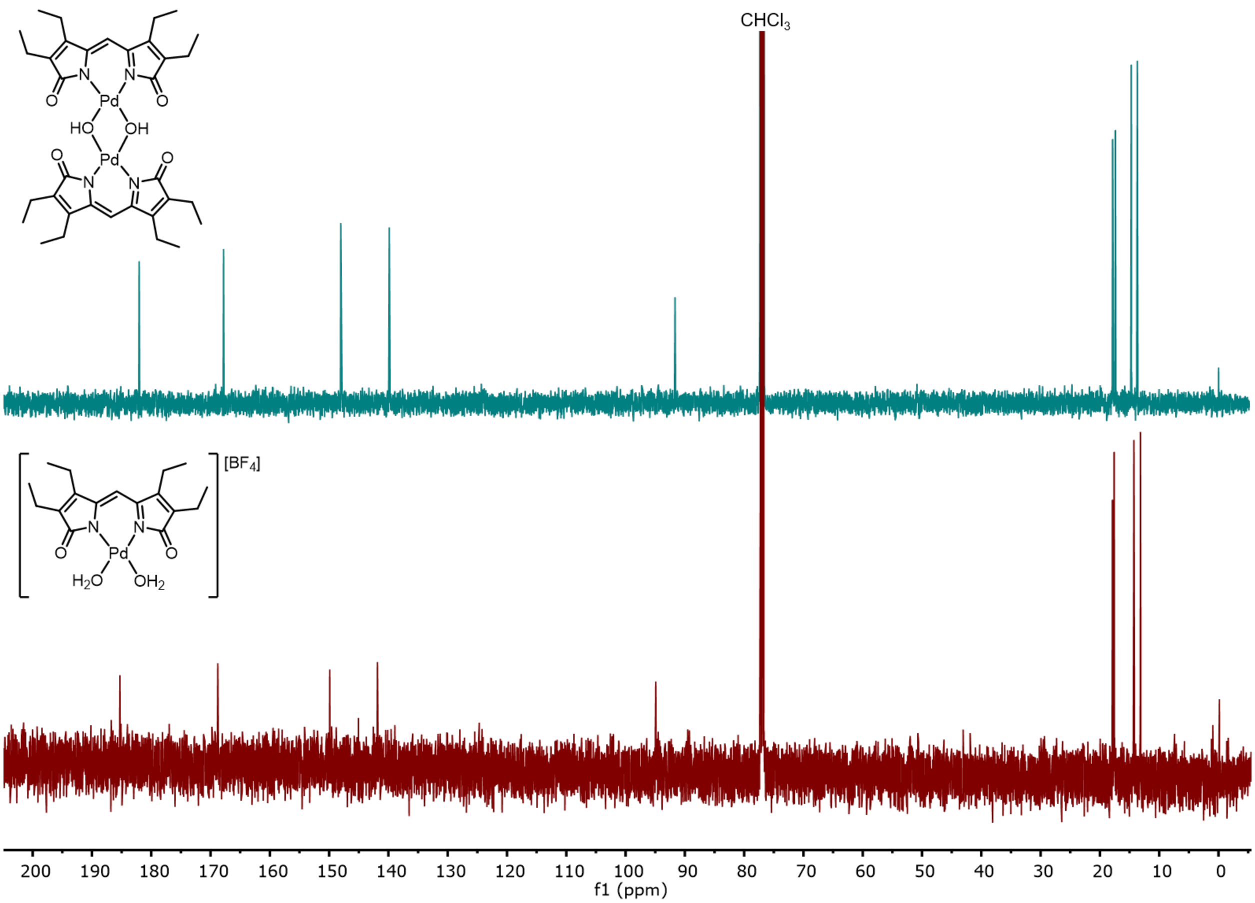


**Figure S2.** $^{13}C$ NMR spectra of [Pd($H_2O$)$_2$(pdp)][$BF_4$] and [Pd(μ-OH)(pdp)]$_2$ complexes in $CDCl_3$.

**Table S2.** Crystal data collection parameters.

| | **[Pd(μ-OH)(pdp)]$_2$** | **[Pd($H_2O$)$_2$(pdp)][$BF_4$]** | **[$CoCp_2$]$_2$[Pd(μ-OH)(pdp)$^{\bullet}$]$_2$** |
|---|---|---|---|
| Molecular Formula | $C_{34}H_{44}N_4O_6Pd_2$ | $C_{17}H_{25}BF_4N_2O_4Pd$ | $C_{28}H_{34}Cl_2CoN_2O_3Pd$ |
| Formula Weight [g·mol$^{-1}$] | 817.53 | 514.60 | 682.80 |
| Temperature [K] | 100.0 | 100.02 | 100.01 |
| Crystal Class | Triclinic | Triclinic | Orthorhombic |
| Space Group | P-1 | P-1 | $P2_12_12_1$ |
| a [Å] | 14.314(8) | 10.520(8) | 12.6676(14) |
| b [Å] | 14.554(9) | 11.155(9) | 16.450(2) |
| c [Å] | 25.246(15) | 11.385(13) | 27.061(4) |
| α [°] | 89.03(2) | 119.28(2) | 90 |
| β [°] | 83.04(2) | 90.02(3) | 90 |
| γ [°] | 70.66(2) | 115.95(2) | 90 |
| Volume [Å$^3$] | 4924(5) | 1007.9(16) | 5639.2(12) |
| Z | 6 | 2 | 8 |
| $\rho_{calc}$ [g·cm$^3$] | 1.654 | 1.696 | 1.608 |
| $\mu$ [mm$^{-1}$] | 1.147 | 0.983 | 1.448 |
| F(000) | 2496.0 | 520.0 | 2776.0 |
| Crystal Size [mm] | 0.29 x 0.06 x 0.06 | 0.168 x 0.206 x 0.306 | 0.111 x 0.076 x 0.074 |
| Measured Reflections | 97197 | 20139 | 87129 |
| Independent Reflections, I > 2σ[*I*] | 20365 | 3807 | 9952 |
| $R_{int}$ | 0.0481 | 0.0166 | 0.1651 |
| Goodness-of-fit on F$^2$ | 1.009 | 1.067 | 1.067 |
| Final *R* indexes for *I* > 2σ[*I*] | $^aR_1$ = 0.0323, $^bwR_2$ = 0.0642 | $^aR_1$ = 0.0254, $^bwR_2$ = 0.0575 | $^aR_1$ = 0.0532, $^bwR_2$ = 0.1073 |
| Final *R* for all data | $^aR_1$ = 0.0583, $^bwR_2$ = 0.0737 | $^aR_1$ = 0.0256, $^bwR_2$ = 0.0577 | $^aR_1$ = 0.1033, $^bwR_2$ = 0.1277 |
| Peak/hole [e·Å$^{-3}$] | 0.98/-0.58 | 1.79/-0.86 | 1.04/-0.84 |
| CCDC Number | 2077677 | 2077676 | 2077678 |

$^aR_1 = \Sigma[|F_o| - |F_c|]/\Sigma|F_o|$ $^bwR_2 = [\Sigma w(F_o^2 - F_c^2)/\Sigma wF_o^4]^{1/2}$

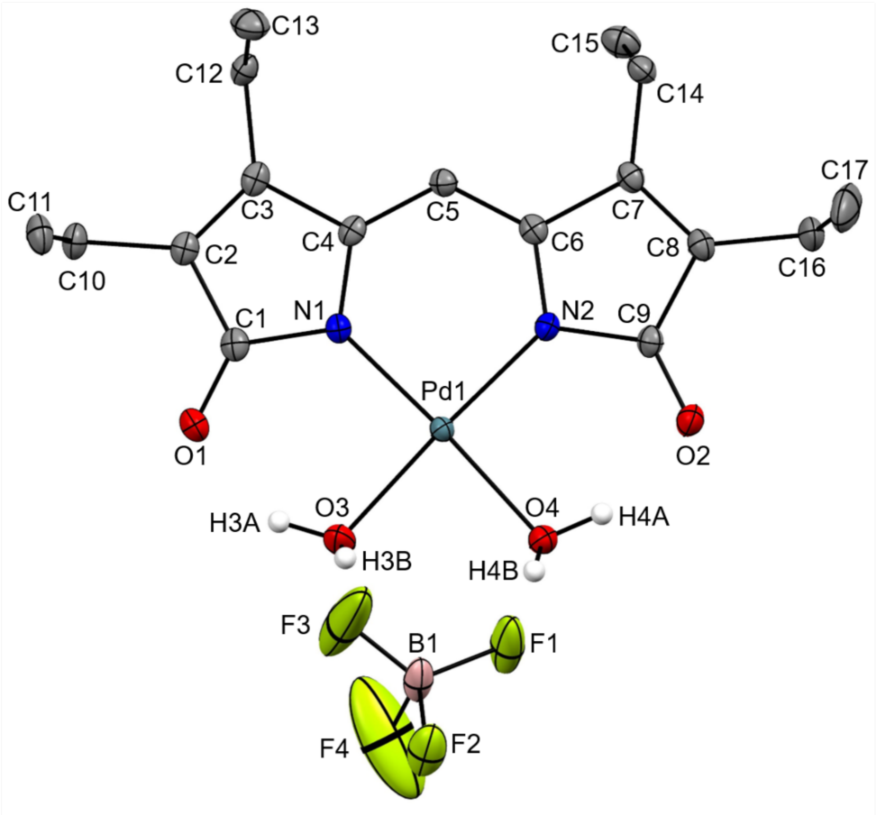


**Figure S3.** Crystal structure of [Pd($H_2O$)$_2$(pdp)][$BF_4$] showing the full labeling scheme. Atoms are displayed as thermal ellipsoids at the 50% probability level. All carbon-bound hydrogens were omitted for clarity.

**Table S3.** Selected bond lengths in the crystal structure of [Pd($H_2O$)$_2$(pdp)][$BF_4$].

| | |
|---|---|
| Pd1 – N1 | 1.984(3) |
| Pd1 – N2 | 2.003(3) |
| Pd1 – O3 | 2.060(3) |
| Pd1 – O4 | 2.044(3) |
| N1 – C1 | 1.419(3) |
| N1 – C4 | 1.347(3) |
| N2 – C6 | 1.353(3) |
| N2 – C9 | 1.410(3) |
| O1 – C1 | 1.226(3) |
| O2 – C2 | 1.223(3) |
| C1 – C2 | 1.481(3) |
| C2 – C3 | 1.342(4) |
| C3 – C4 | 1.494(4) |
| C4 – C5 | 1.398(4) |
| C5 – C6 | 1.380(4) |
| C6 – C7 | 1.499(3) |
| C7 – C8 | 1.335(4) |
| C8 – C9 | 1.495(4) |

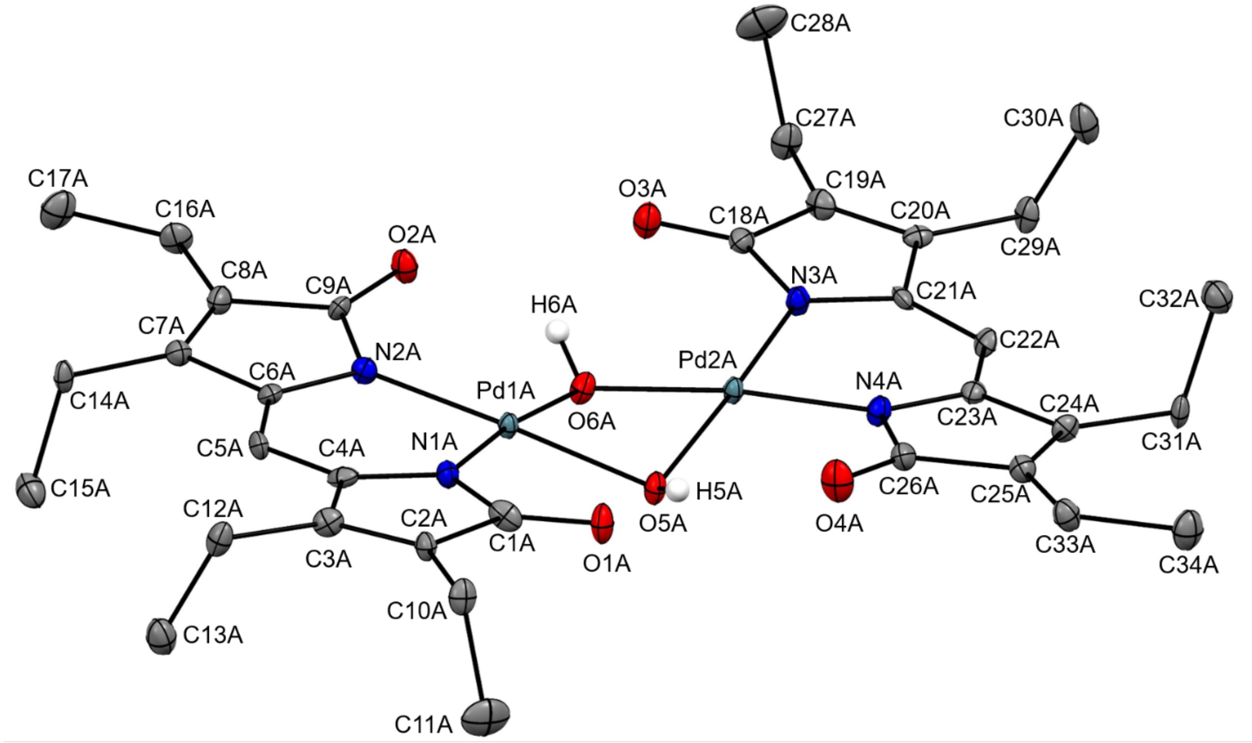


**Figure S4.** Crystal structure of one of the three complexes in the unit cell of $[Pd(\mu\text{-}OH)(pdp)]_2$ showing the full labeling scheme. Atoms are displayed as thermal ellipsoids at the 50% probability level. All carbon-bound hydrogens were omitted for clarity.

**Table S4.** Selected bond lengths in the crystal structures of $[Pd(\mu\text{-}OH)(pdp)]_2$ and $[CoCp_2]_2[Pd(\mu\text{-}OH)(pdp)^{\bullet}]_2$. Elongation of bonds upon reduction are shown in red, and contraction of bonds are shown in green.

| Bond | $[Pd_2(\mu\text{-}OH)_2(pdp)_2]$ A | B | C | $[CoCp_2]_2[Pd(\mu\text{-}OH)(pdp)^{\bullet}]_2$ |
|---|---|---|---|---|
| Pd1-N1 | 2.003(3) | 1.991(3) | 1.994(3) | 2.030(10) |
| Pd1-N2 | 1.989(3) | 1.987(3) | 1.979(3) | 2.018(10) |
| Pd2-N3 | 1.992(3) | 1.988(3) | 1.995(3) | 2.025(10) |
| Pd2-N4 | 1.983(3) | 1.988(3) | 1.981(3) | 1.989(10) |
| Pd1-O5 | 1.993(3) | 1.999(2) | 1.998(3) | 2.036(8) |
| Pd1-O6 | 2.004(3) | 2.008(3) | 2.014(3) | 2.039(8) |
| Pd2-O5 | 2.008(3) | 2.004(3) | 2.001(3) | 2.050(8) |
| Pd2-O6 | 2.009(3) | 2.008(3) | 2.012(3) | 2.017(8) |
| N1-C1 | 1.420(4) | 1.417(4) | 1.417(4) | 1.406(16) |
| N1-C4 | 1.331(4) | 1.336(4) | 1.341(4) | 1.372(15) |
| N2-C6 | 1.327(4) | 1.335(4) | 1.345(4) | 1.393(15) |
| N2-C9 | 1.431(4) | 1.417(4) | 1.418(4) | 1.403(16) |
| N3-C18 | 1.410(4) | 1.424(4) | 1.416(4) | 1.388(15) |
| N3-C21 | 1.342(4) | 1.338(4) | 1.340(4) | 1.375(18) |
| N4-C23 | 1.338(4) | 1.343(4) | 1.332(4) | 1.420(16) |
| N4-C26 | 1.422(4) | 1.413(4) | 1.430(4) | 1.396(15) |
| O1-C1 | 1.197(4) | 1.206(4) | 1.200(4) | 1.240(14) |
| O2-C9 | 1.205(4) | 1.205(4) | 1.208(4) | 1.244(14) |
| O3-C18 | 1.209(4) | 1.199(4) | 1.201(4) | 1.245(15) |
| O4-C26 | 1.207(4) | 1.213(4) | 1.201(4) | 1.263(15) |
| C1-C2 | 1.493(4) | 1.495(4) | 1.493(4) | 1.511(17) |
| C2-C3 | 1.338(4) | 1.334(4) | 1.330(5) | 1.355(17) |
| C3-C4 | 1.487(4) | 1.485(4) | 1.491(4) | 1.462(17) |
| C4-C5 | 1.377(4) | 1.381(4) | 1.374(4) | 1.360(17) |
| C5-C6 | 1.385(4) | 1.387(4) | 1.387(4) | 1.416(16) |
| C6-C7 | 1.490(4) | 1.484(4) | 1.487(4) | 1.436(17) |
| C7-C8 | 1.335(4) | 1.346(4) | 1.335(4) | 1.354(17) |
| C8-C9 | 1.497(4) | 1.490(4) | 1.482(4) | 1.486(17) |
| C18-C19 | 1.492(4) | 1.490(4) | 1.498(4) | 1.483(18) |
| C19-C20 | 1.332(4) | 1.332(4) | 1.333(4) | 1.363(18) |
| C20-C21 | 1.496(4) | 1.492(4) | 1.491(4) | 1.490(17) |
| C21-C22 | 1.385(4) | 1.380(4) | 1.377(4) | 1.374(17) |
| C22-C23 | 1.388(4) | 1.382(4) | 1.385(4) | 1.376(17) |
| C23-C24 | 1.484(4) | 1.491(4) | 1.487(4) | 1.443(18) |
| C24-C25 | 1.342(4) | 1.346(4) | 1.331(4) | 1.361(18) |
| C25-C26 | 1.484(4) | 1.486(4) | 1.483(5) | 1.469(18) |

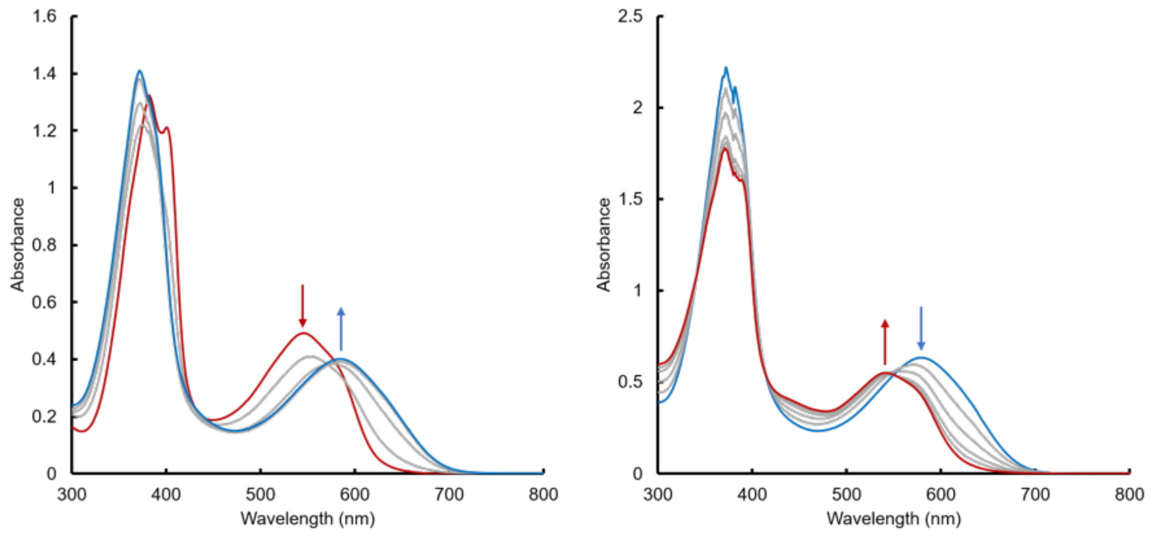


**Figure S5.** Titration of $[Pd(H_2O)_2(pdp)][BF_4]$ (75 µM, $CH_2Cl_2$) with $Et_3N$ (2.0 equiv) to generate [Pd(µ-OH)(pdp)]$_2$ (left) and titration [Pd(µ-OH)(pdp)]$_2$ (51 µM, 95:5 v/v $CH_2Cl_2$:MeOH) with TFA (2.0 equiv) to generate $[Pd(H_2O)_2(pdp)][BF_4]$.

**Table S5.** Comparison of experimental and calculated bond lengths for $[Pd(H_2O)_2(pdp)]^+$ and [[Pd(µ-OH)(pdp)]$_2$.

| | **$[Pd(H_2O)_2(pdp)]^+$ (exp)** | **$[Pd(H_2O)_2(pdp)]^+$ (calc.)** | **[[Pd(µ-OH)(pdp)]$_2$ (exp)** | **[[Pd(µ-OH)(pdp)]$_2$ (calc., $D_{2h}$)** |
|---|---|---|---|---|
| Pd1 – N1 | 1.984(3) | 2.020 | 2.003(3) | 2.026 |
| Pd1 – O3(O5) | 2.060(3) | 2.113 | 2.008(3) | 2.031 |
| N1 – C1 | 1.419(3) | 1.429 | 1.420(4) | 1.429 |
| N1 – C4 | 1.347(3) | 1.346 | 1.331(4) | 1.342 |
| C1 – O1 | 1.226(3) | 1.221 | 1.197(4) | 1.216 |
| C1 – C2 | 1.481(3) | 1.490 | 1.493(4) | 1.499 |
| C2 – C3 | 1.342(4) | 1.341 | 1.338(4) | 1.340 |
| C3 – C4 | 1.494(4) | 1.479 | 1.487(4) | 1.479 |
| C4 – C5 | 1.398(4) | 1.391 | 1.377(4) | 1.395 |

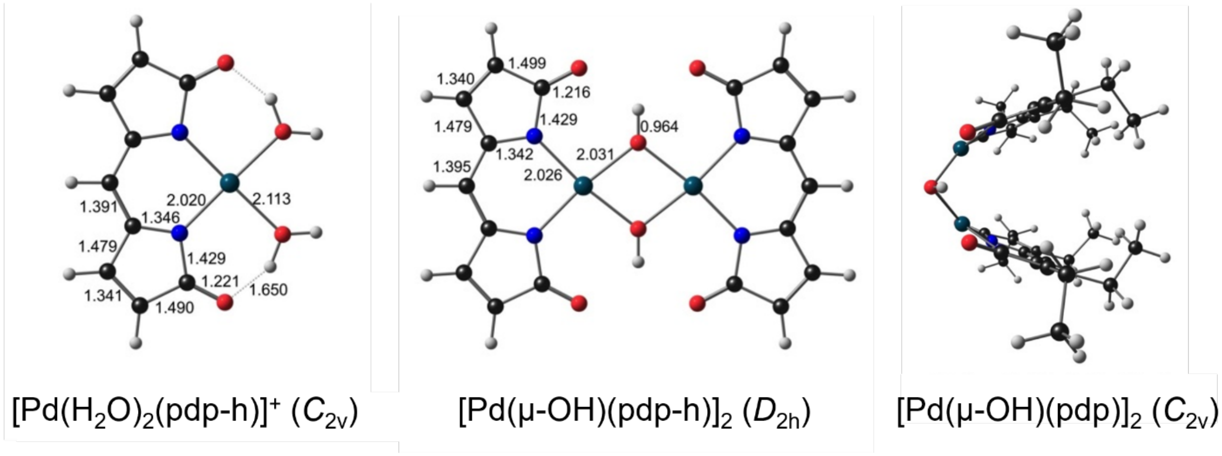


**Figure S6.** Selected OLYP/STO-TZ2P optimized geometries (Å).

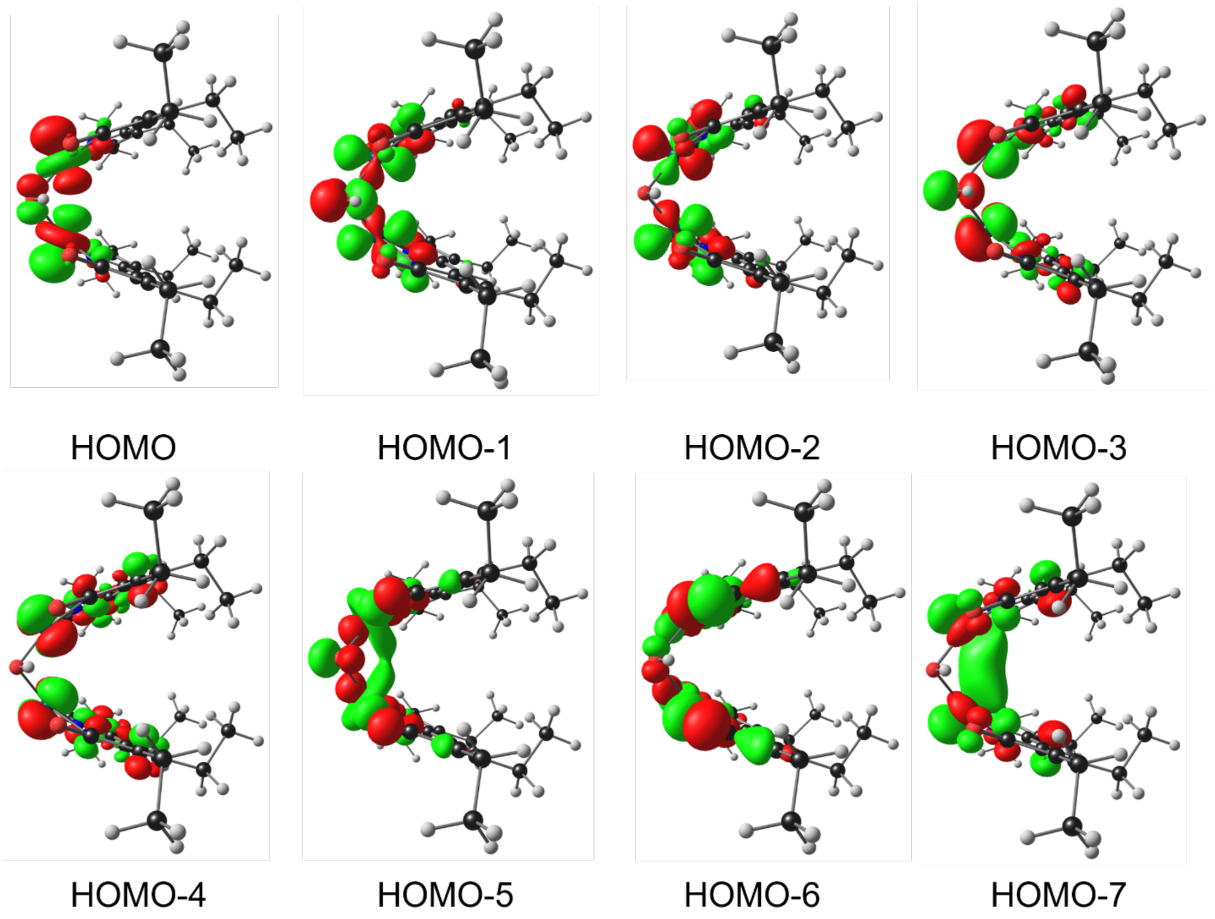


**Figure S7.** Frontier Kohn-Sham MOs of $[Pd(\mu\text{-}OH)(pdp)]_2$ ($C_{2v}$) showing bonding (HOMO-5, $d_{xy} - d_{xy}$; HOMO-7, $d_{z2} - d_{z2}$) and antibonding (HOMO, $d_{z2} - d_{z2}$) $d^8$-$d^8$ interactions between the orbitals of the $Pd^{II}$ centers.

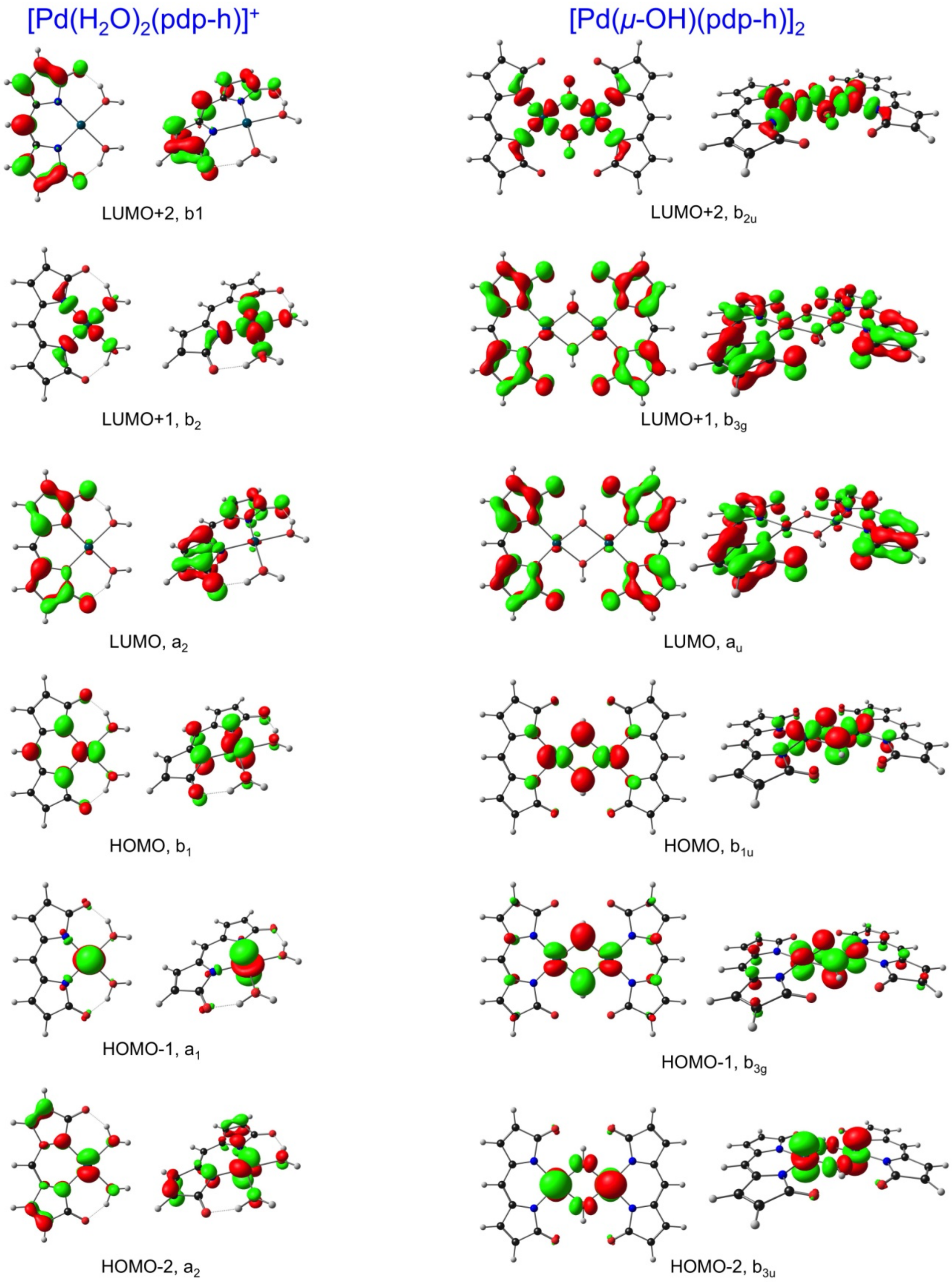


**Figure S8.** Frontier Kohn-Sham MOs of the simplified model complexes $[Pd(H_2O)_2(pdp\text{-}h)]^+$ ($C_{2v}$) and $[Pd(\mu\text{-}OH)(pdp\text{-}h)]_2$ ($D_{2h}$). Top and side views are depicted for each MO.

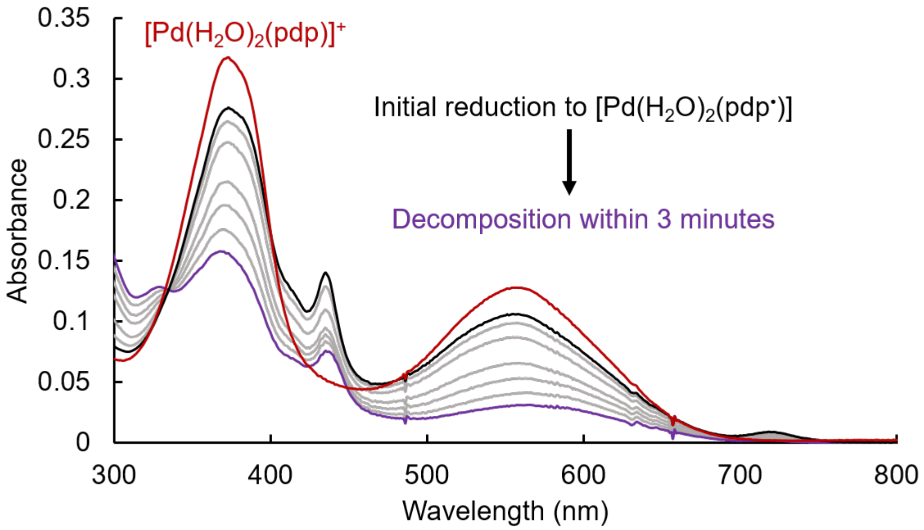


**Figure 9.** Spectral changes observed upon reduction of $[Pd(H_2O)_2(pdp)]^+$ (red trace) (0.1 M $(NBu_4)(PF_6)$ in DMF) by controlled potential electrolysis at -0.7 V. Initial growth of bands at 437 and 720 nm hint at the formation of a ligand-based radical product (black trace); however, the absorption of this new species and the parent complex rapidly decreased over time indicating rapid decomposition (purple trace).

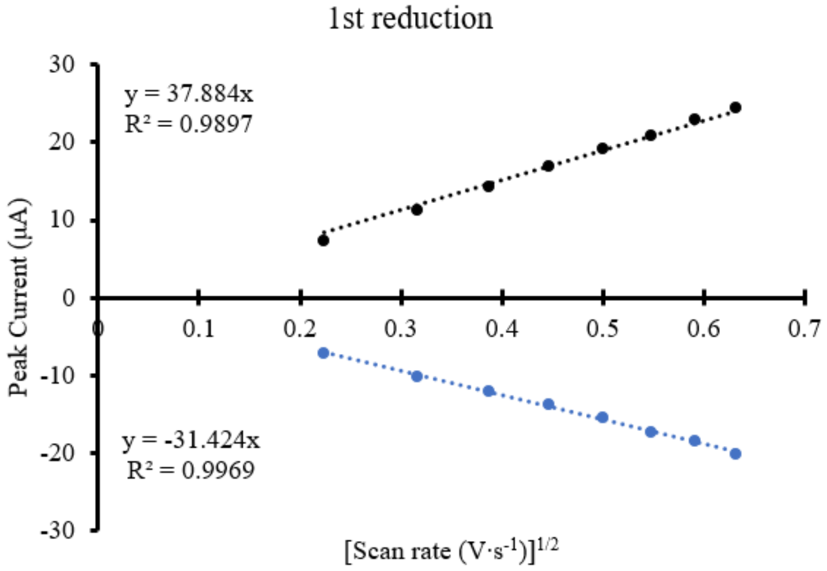


**Figure S10.** Plot of measured peak current vs square root of applied scan rate for the first reduction event of $[Pd(\mu\text{-}OH)(pdp)]_2$.

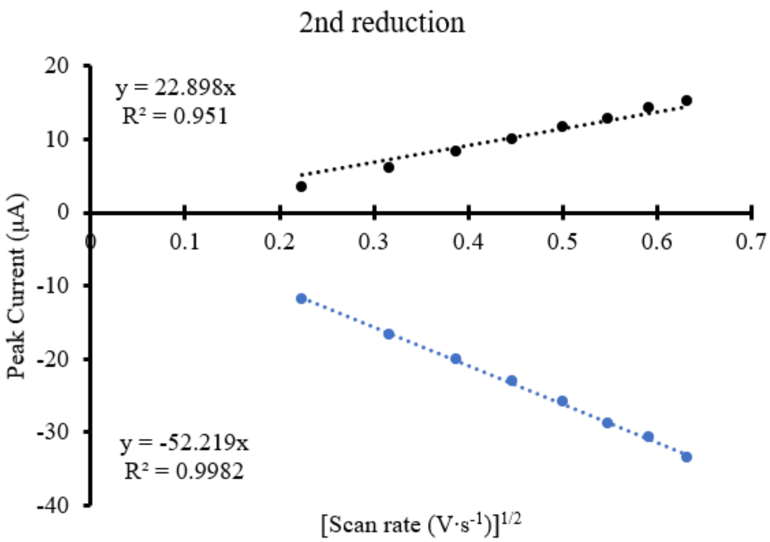


**Figure S11.** Plot of measured peak current vs square root of applied scan rate for the second reduction event of $[Pd(\mu\text{-}OH)(pdp)]_2$.

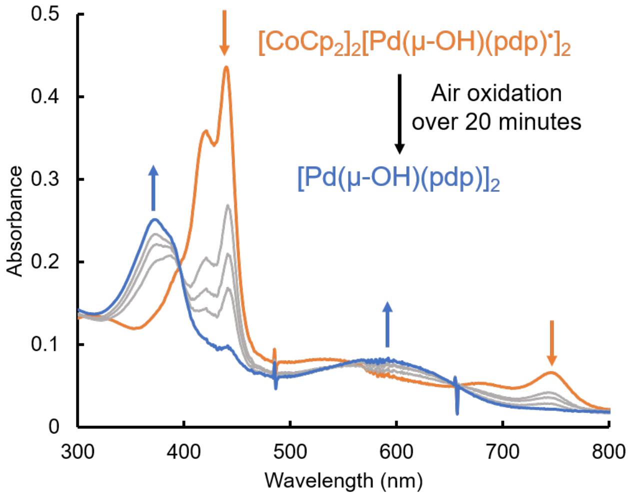


**Figure S12.** Oxidation of $[CoCp_2]_2[Pd(\mu\text{-}OH)(pdp)^{\bullet}]_2$ back to $[Pd(\mu\text{-}OH)(pdp)]_2$ with partial decomposition upon exposure to atmospheric oxygen over twenty minutes.

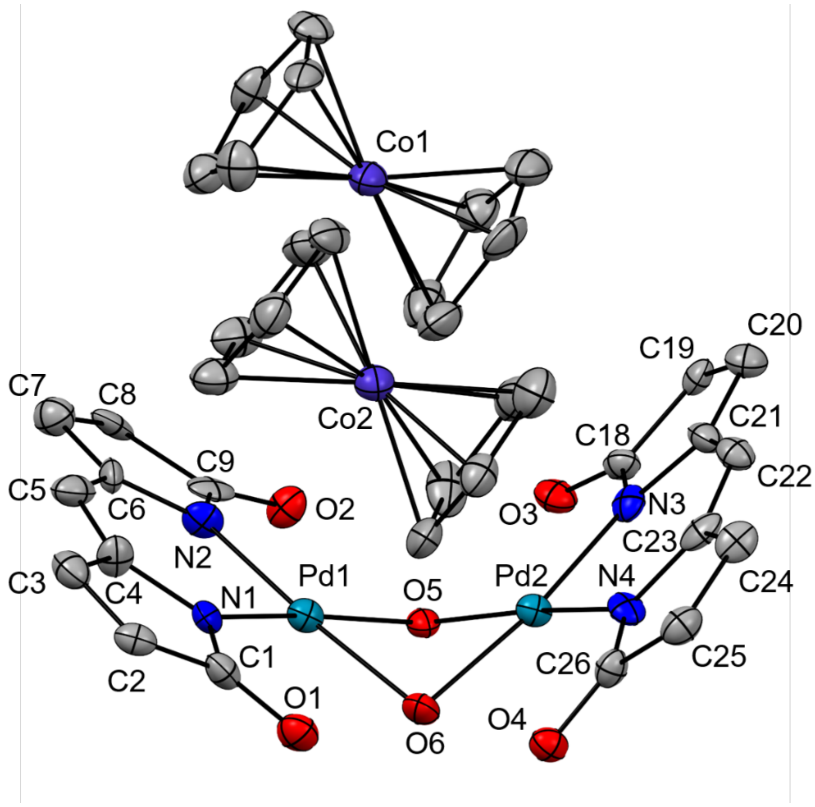


**Figure S13.** Crystal structure of $[CoCp_2]_2[Pd(\mu\text{-}OH)(pdp)^{\bullet}]_2$ showing full labeling of the dipyrrindione scaffolds. Atoms are displayed as thermal ellipsoids at the 50% probability level. All hydrogens and ethyl substituents were omitted for clarity.

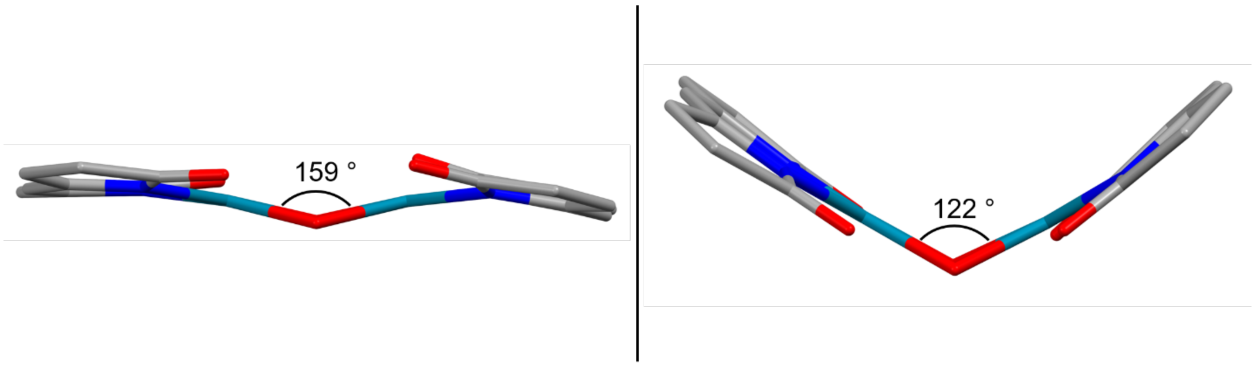


**Figure S14.** Side views of $[Pd(\mu\text{-}OH)(pdp)]_2$ (left) and $[Pd(\mu\text{-}OH)(pdp)^{\bullet}]_2^{2-}$ (right) showing the change in fold angle between $Pd^{II}$ coordination planes upon ligand reduction.

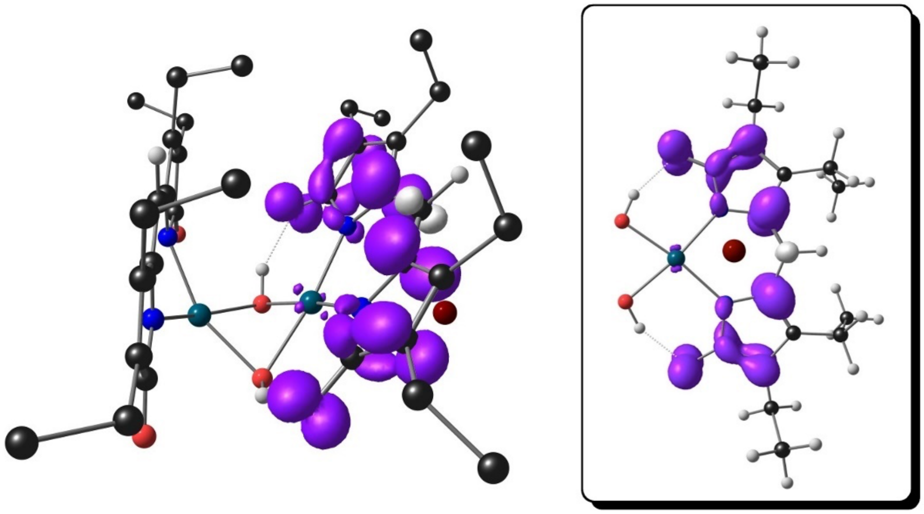

**Figure S15**. OLYP-D3/TZ2P spin density plot for Cs[$Pd_2$(μ-OH)$_2$(pdp)(pdp)$^•$]. Inset: detail of the spin-bearing half of the molecule. The $Cs^+$ atom is shown in maroon.

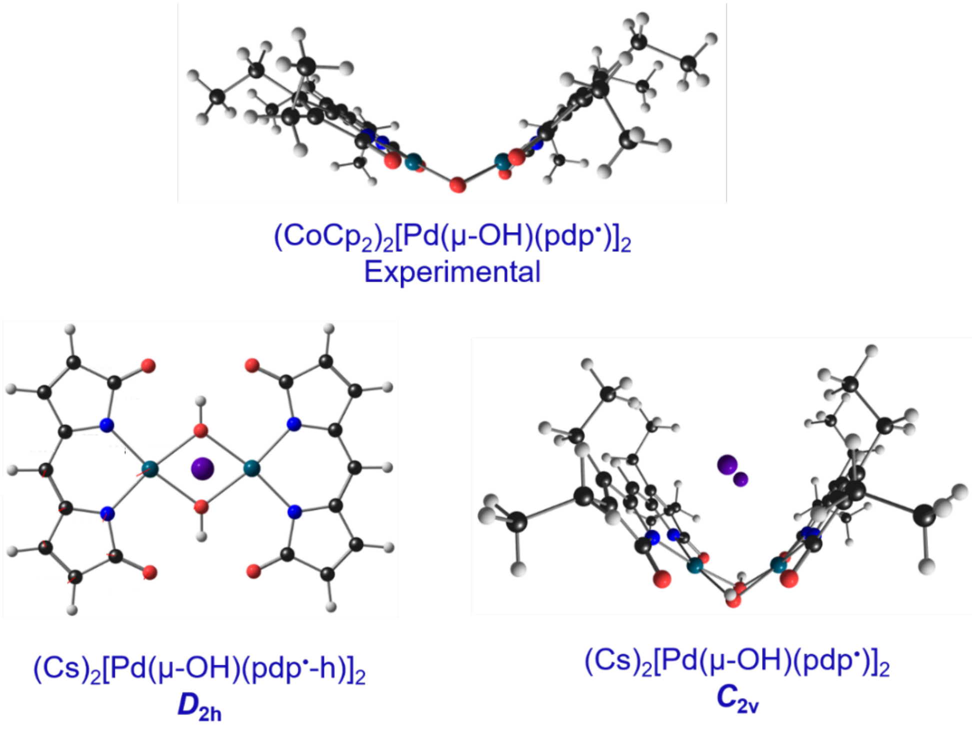


**Figure S16.** Comparison of experimental and calculated structures of dianion [Pd(μ-OH)(pdp)$^•$]$_2^{2-}$: (top) experimental geometry obtained from crystal structure (cobaltocenium cations omitted for clarity), (bottom right) $C_{2v}$ symmetry with two $Cs^+$ ions (overlapping in side view), and (bottom left) constrained $D_{2h}$ symmetry with two $Cs^+$ ions (overlapping in top view). The angle between the pdp ligand planes is 101.6º in the experimental structure, 69.8º in the $C_{2v}$ calculated structure, and 180º in the $D_{2h}$ calculated structure.

**Table S6.** Comparison of experimental and calculated bond lengths for $[[Pd(\mu\text{-}OH)(pdp)^{\bullet}]_2]^{2-}$.

| | **[CoCp₂]₂[[Pd(μ-OH)(pdp)•]₂ (exp.)** | **Cs₂[[Pd(μ-OH)(pdp)•]₂ (calc., *C*₂v)** |
|---|---|---|
| Pd1 – N1 | 2.030(10) | 2.066 |
| Pd1 – O5 | 2.036(8) | 2.085 |
| N1 – C1 | 1.406(16) | 1.403 |
| N1 – C4 | 1.372(15) | 1.370 |
| C1 – O1 | 1.240(14) | 1.237 |
| C1 – C2 | 1.511(17) | 1.486 |
| C2 – C3 | 1.355(17) | 1.365 |
| C3 – C4 | 1.462(17) | 1.458 |
| C4 – C5 | 1.360(17) | 1.394 |

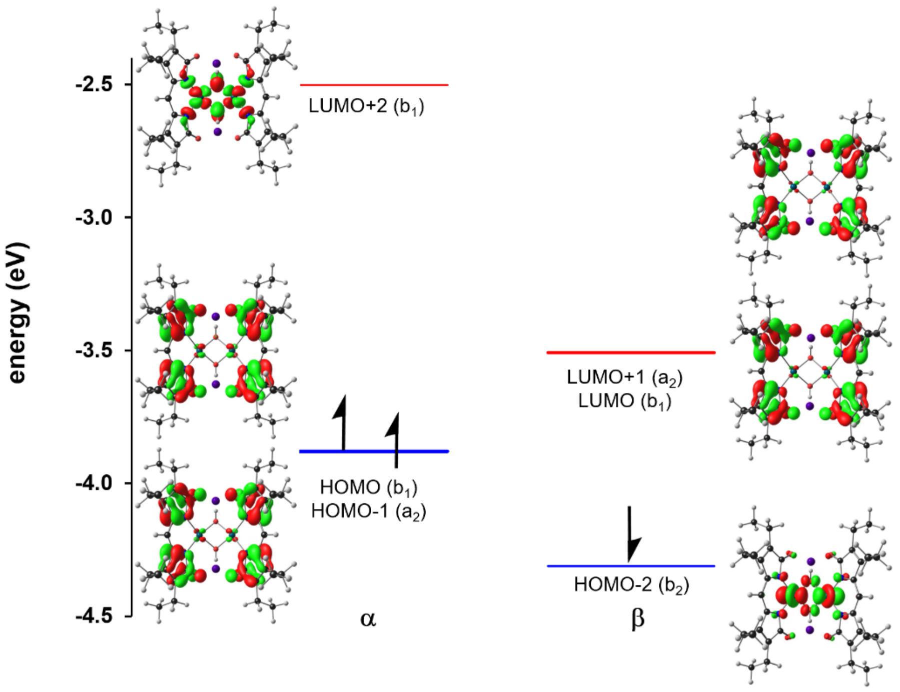


**Figure S17.** Frontier Kohn-Sham MOs of $Cs_2[Pd(\mu\text{-}OH)(pdp)^{\bullet}]_2$ ($C_{2v}$).

**Table S7.** Mulliken spin populations in the DFT structural models of $[Pd(\mu\text{-}OH)(pdp)^{\bullet}]_2^{2-}$. The spin populations have been normalized to ensure the total one-electron spin population of 1. The corresponding atom numbering scheme is shown in Fig. S18.

| Structural model | Pd | N | O | C1 | C2 | C3 | C4 | C5 |
|---|---|---|---|---|---|---|---|---|
| $C_{2v}$: $Cs_2[Pd(\mu\text{-}OH)(pdp\text{-}h)^{\bullet}]_2$ | 0.004 | 0.015 | 0.140 | 0.050 | 0.135 | 0.011 | 0.187 | 0.080 |
| $D_{2h}$: $Cs_2[Pd(\mu\text{-}OH)(pdp)^{\bullet}]_2$ | 0.012 | 0.018 | 0.153 | 0.048 | 0.123 | 0.010 | 0.182 | 0.080 |

**Calculation of magnetic dipole interaction in dianionic complexes**

To calculate the magnetic dipole interaction, each unpaired electron was represented by a set of 14 points coinciding with the position of the ligand atoms and the Pd(II) ion (Fig. S18) and with spin populations calculated by DFT (Table S7).

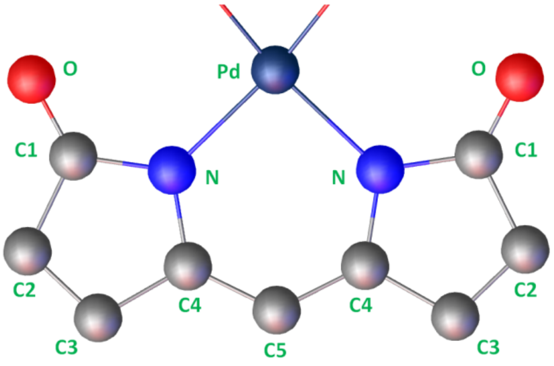


**Figure S18.** Atom numbering used in Table S5.

To calculate the dipole interaction parameters for the crystal structure, we used the spin populations of the $C_{2v}$ calculated structure because it was closer to the experimental one with regards to the angle between the ligand planes. Although the actual spin density distributions in these two structures are probably somewhat different, this difference is expected to be insignificant because the differences between spin density distributions in the calculated structures encompassing a wide range of the interplane angles were small, and the distributions were generally similar. Table S6 shows the magnetic dipole interactions calculated for different structural models in comparison with the experimental tensor obtained by EPR. The D-tensor estimated for the conformation found in the crystal structure is in very good agreement with the experimental one, indicating that such a conformation is apparently preserved in solution. These calculations also allow assignment of the principal directions of the dipole interaction to molecular axes: specifically, axis *z* coincides with the $C_2$ symmetry axis of the dimeric complex, axis *x* points in the Pd-to-Pd direction, and axis *y* is parallel to the line joining the bridging oxygen atoms (see Fig. S19).

**Table S8.** Magnetic dipole interaction tensors in $[Pd(\mu\text{-}OH)(pdp)^{\bullet}]_2^{2-}$ obtained by EPR measurements and by calculations based on the crystal structure and optimized DFT structures.

| Dipole interaction tensor source: EPR experiment or calculation based on the crystal structure and DFT structural models. | Angle between ligand planes | $(D_x, D_y, D_z)$ |
|---|---|---|
| **EPR experiment** | | **(-330, 130, 200) MHz** |
| DFT $D_{2h}$: $Cs_2[Pd(\mu\text{-}OH)(pdp\text{-}h)^{\bullet}]_2$ | 180° | (-292.2, 113.3, 178.9) MHz |
| crystal structure (with spin density of $C_{2v}$ model) | 101.6° | (-331, 131.4, 199.6) MHz |
| DFT $C_{2v}$: $Cs_2[Pd(\mu\text{-}OH)(pdp)^{\bullet}]_2$ | 69.8° | (-394.8, 157.8, 237.0) MHz |

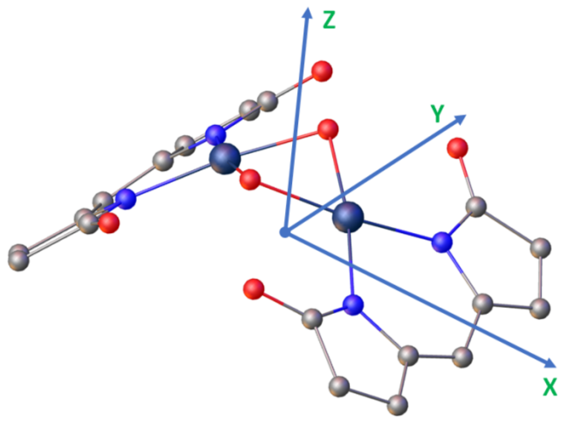


**Figure S19.** Principal axes of the dipole interaction tensor.

### Exchange interaction in the dianionic complex $[Pd(\mu\text{-}OH)(pdp)^{\bullet}]_2^{2-}$

To estimate the exchange interaction in the dianionic species, variable-temperature EPR measurements were performed. The red circles in Fig. S20 show the temperature dependence of the electron spin echo (ESE) signal at the maximum of the $K_a$-band field sweep spectrum shown by trace 2 in Fig. 9 of the main text. Solid lines show the theoretical signal intensities (which are directly proportional to the population differences in the triplet manifold) calculated for several $J$-values (assuming the $-2JS_1S_2$ parametrization of the exchange term in the spin Hamiltonian). The experimental temperature dependence is closely reproduced by the red line calculated for $J$ = -2.5 K (-1.75 cm$^{-1}$), with the possible error limits at about ±0.5 K (0.35 cm$^{-1}$).

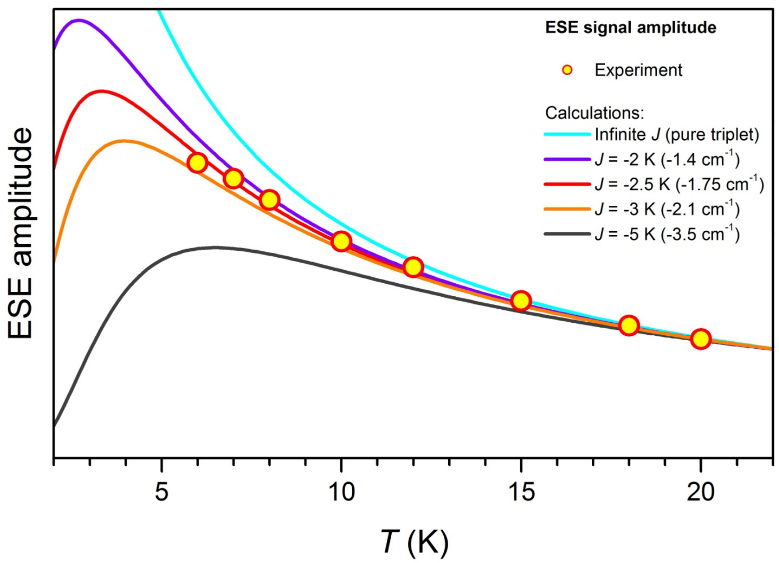


**Figure S20.** ESE signal temperature dependence for the dianionic diradical complex $[Pd(\mu\text{-}OH)(pdp)^{\bullet}]_2^{2-}$. Experimental measurements are shown as red circles. The colored solid lines are the scaled population differences in the triplet manifold calculated for different *J*-values. The experimental measurements have been performed at the mw frequency of 34.576 GHz and $B_o$ = 1.23 T (maximum of the field sweep spectrum) using the primary echo sequence with $\tau$ = 200 ns.

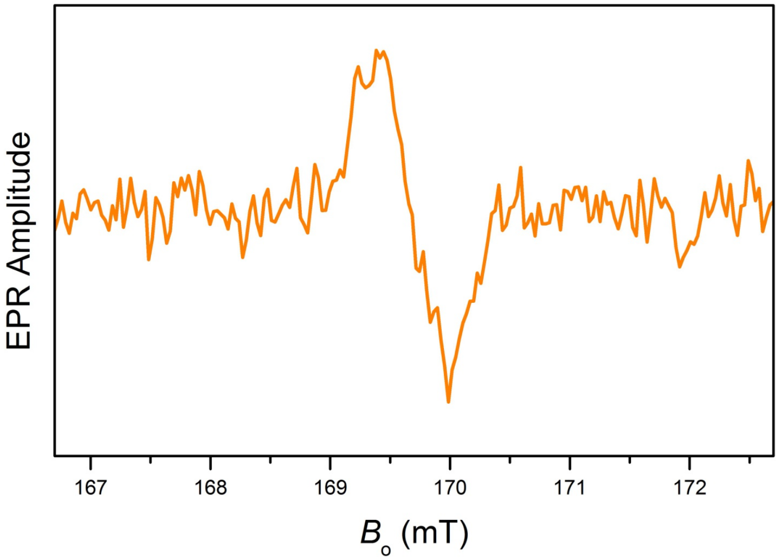


**Figure S21.** Half-field ($\Delta M$ = 2) EPR transition detected for the dianionic diradical complex $[Pd(\mu\text{-}OH)(pdp)^{\bullet}]_2^{2-}$. Experimental conditions: mw frequency, 9.472 GHz, mw power, 2 mW; field modulation amplitude, 0.5 mT; temperature, 77 K.

**Optimized cartesian coordinates (Å)**

All compounds were optimized using the STO-TZ2P basis set, all electron, OLYP-D3 functional.

## 1. $[Pd(H_2O)_2(pdp\text{-}h)]^+$, $C_{2v}$, q = +1, $S$ = 0

| | | | |
|---|---|---|---|
| Pd | 0.000000000 | 0.000000000 | 0.888733000 |
| C | 0.000000000 | 0.000000000 | -2.507816000 |
| C | 0.000000000 | 1.230522000 | -1.859577000 |
| C | 0.000000000 | 2.524294000 | -2.575969000 |
| C | 0.000000000 | 2.848235000 | -0.317540000 |
| C | 0.000000000 | 3.499435000 | -1.657438000 |
| C | 0.000000000 | -1.230522000 | -1.859577000 |
| C | 0.000000000 | -2.524294000 | -2.575969000 |
| C | 0.000000000 | -2.848235000 | -0.317540000 |
| C | 0.000000000 | -3.499435000 | -1.657438000 |
| H | 0.000000000 | 0.000000000 | -3.592638000 |
| H | 0.000000000 | 1.412920000 | 3.365565000 |
| H | 0.000000000 | 2.393844000 | 2.052014000 |
| H | 0.000000000 | 2.601023000 | -3.656347000 |
| H | 0.000000000 | 4.574898000 | -1.777216000 |
| H | 0.000000000 | -1.412920000 | 3.365565000 |
| H | 0.000000000 | -2.393844000 | 2.052014000 |
| H | 0.000000000 | -2.601023000 | -3.656347000 |
| H | 0.000000000 | -4.574898000 | -1.777216000 |
| N | 0.000000000 | 1.435173000 | -0.529937000 |
| N | 0.000000000 | -1.435173000 | -0.529937000 |
| O | 0.000000000 | 1.469546000 | 2.405975000 |
| O | 0.000000000 | 3.415761000 | 0.764019000 |
| O | 0.000000000 | -1.469546000 | 2.405975000 |
| O | 0.000000000 | -3.415761000 | 0.764019000 |

## 2. [Pd(μ-OH)(pdp-h)]$_2$, $D_{2h}$, q = 0, $S$ = 0

| | | | |
|---|---|---|---|
| Pd | -1.598227000 | 0.000000000 | 0.000000000 |
| Pd | 1.598227000 | 0.000000000 | 0.000000000 |
| C | 2.797888000 | 2.858428000 | 0.000000000 |
| C | 2.797888000 | -2.858428000 | 0.000000000 |
| C | 4.148812000 | 3.506997000 | 0.000000000 |
| C | 4.148812000 | -3.506997000 | 0.000000000 |
| C | 4.343032000 | 1.238044000 | 0.000000000 |
| C | 4.343032000 | -1.238044000 | 0.000000000 |
| C | 4.985213000 | 0.000000000 | 0.000000000 |
| C | 5.065304000 | 2.529196000 | 0.000000000 |
| C | 5.065304000 | -2.529196000 | 0.000000000 |
| C | -2.797888000 | 2.858428000 | 0.000000000 |
| C | -2.797888000 | -2.858428000 | 0.000000000 |
| C | -4.148812000 | 3.506997000 | 0.000000000 |
| C | -4.148812000 | -3.506997000 | 0.000000000 |
| C | -4.343032000 | 1.238044000 | 0.000000000 |
| C | -4.343032000 | -1.238044000 | 0.000000000 |
| C | -4.985213000 | 0.000000000 | 0.000000000 |
| C | -5.065304000 | 2.529196000 | 0.000000000 |
| C | -5.065304000 | -2.529196000 | 0.000000000 |
| H | 0.000000000 | 2.217626000 | 0.000000000 |
| H | 0.000000000 | -2.217626000 | 0.000000000 |
| H | 4.275627000 | 4.581431000 | 0.000000000 |
| H | 4.275627000 | -4.581431000 | 0.000000000 |
| H | 6.070782000 | 0.000000000 | 0.000000000 |
| H | 6.146326000 | 2.603651000 | 0.000000000 |
| H | 6.146326000 | -2.603651000 | 0.000000000 |

| | | | |
|---|---|---|---|
| H | -4.275627000 | 4.581431000 | 0.000000000 |
| H | -4.275627000 | -4.581431000 | 0.000000000 |
| H | -6.070782000 | 0.000000000 | 0.000000000 |
| H | -6.146326000 | 2.603651000 | 0.000000000 |
| H | -6.146326000 | -2.603651000 | 0.000000000 |
| N | 3.016799000 | 1.446110000 | 0.000000000 |
| N | 3.016799000 | -1.446110000 | 0.000000000 |
| N | -3.016799000 | 1.446110000 | 0.000000000 |
| N | -3.016799000 | -1.446110000 | 0.000000000 |
| O | 0.000000000 | 1.253383000 | 0.000000000 |
| O | 0.000000000 | -1.253383000 | 0.000000000 |
| O | 1.720695000 | 3.422809000 | 0.000000000 |
| O | 1.720695000 | -3.422809000 | 0.000000000 |
| O | -1.720695000 | 3.422809000 | 0.000000000 |
| O | -1.720695000 | -3.422809000 | 0.000000000 |

**3. [Pd(μ-OH)(pdp)]$_2$, $C_{2v}$, q = 0, S = 0**

| | | | |
|---|---|---|---|
| Pd | 1.342440000 | 0.000000000 | -2.923288000 |
| Pd | -1.342440000 | 0.000000000 | -2.923288000 |
| C | 2.013313000 | 2.855937000 | -1.885742000 |
| C | 2.013313000 | -2.855937000 | -1.885742000 |
| C | 2.121443000 | 2.321996000 | 2.669051000 |
| C | 2.121443000 | -2.321996000 | 2.669051000 |
| C | 2.363078000 | 4.983504000 | -0.388943000 |
| C | 2.363078000 | -4.983504000 | -0.388943000 |
| C | 2.415947000 | 3.509922000 | -0.589933000 |
| C | 2.415947000 | -3.509922000 | -0.589933000 |
| C | 2.633332000 | 1.241071000 | -0.487133000 |
| C | 2.633332000 | -1.241071000 | -0.487133000 |

| | | | |
|---|---|---|---|
| C | 2.805461000 | 2.528215000 | 0.246378000 |
| C | 2.805461000 | -2.528215000 | 0.246378000 |
| C | 2.870677000 | 0.000000000 | 0.101684000 |
| C | 3.250468000 | 2.638896000 | 1.669849000 |
| C | 3.250468000 | -2.638896000 | 1.669849000 |
| C | 3.689017000 | 5.670706000 | -0.764843000 |
| C | 3.689017000 | -5.670706000 | -0.764843000 |
| C | -2.013313000 | 2.855937000 | -1.885742000 |
| C | -2.013313000 | -2.855937000 | -1.885742000 |
| C | -2.121443000 | 2.321996000 | 2.669051000 |
| C | -2.121443000 | -2.321996000 | 2.669051000 |
| C | -2.363078000 | 4.983504000 | -0.388943000 |
| C | -2.363078000 | -4.983504000 | -0.388943000 |
| C | -2.415947000 | 3.509922000 | -0.589933000 |
| C | -2.415947000 | -3.509922000 | -0.589933000 |
| C | -2.633332000 | 1.241071000 | -0.487133000 |
| C | -2.633332000 | -1.241071000 | -0.487133000 |
| C | -2.805461000 | 2.528215000 | 0.246378000 |
| C | -2.805461000 | -2.528215000 | 0.246378000 |
| C | -2.870677000 | 0.000000000 | 0.101684000 |
| C | -3.250468000 | 2.638896000 | 1.669849000 |
| C | -3.250468000 | -2.638896000 | 1.669849000 |
| C | -3.689017000 | 5.670706000 | -0.764843000 |
| C | -3.689017000 | -5.670706000 | -0.764843000 |
| H | 0.000000000 | 2.142962000 | -3.625238000 |
| H | 0.000000000 | -2.142962000 | -3.625238000 |
| H | 1.297784000 | 3.032015000 | 2.547921000 |
| H | 1.297784000 | -3.032015000 | 2.547921000 |
| H | 1.560127000 | 5.389022000 | -1.014908000 |
| H | 1.560127000 | -5.389022000 | -1.014908000 |

| | | | |
|---|---|---|---|
| H | 1.724261000 | 1.316273000 | 2.504707000 |
| H | 1.724261000 | -1.316273000 | 2.504707000 |
| H | 2.122997000 | 5.204324000 | 0.658134000 |
| H | 2.122997000 | -5.204324000 | 0.658134000 |
| H | 2.490828000 | 2.387789000 | 3.698994000 |
| H | 2.490828000 | -2.387789000 | 3.698994000 |
| H | 3.234145000 | 0.000000000 | 1.120029000 |
| H | 3.611500000 | 6.753701000 | -0.617040000 |
| H | 3.611500000 | -6.753701000 | -0.617040000 |
| H | 3.615924000 | 3.654719000 | 1.851613000 |
| H | 3.615924000 | -3.654719000 | 1.851613000 |
| H | 3.932311000 | 5.477775000 | -1.814220000 |
| H | 3.932311000 | -5.477775000 | -1.814220000 |
| H | 4.095073000 | 1.962587000 | 1.850981000 |
| H | 4.095073000 | -1.962587000 | 1.850981000 |
| H | 4.507756000 | 5.290022000 | -0.144472000 |
| H | 4.507756000 | -5.290022000 | -0.144472000 |
| H | -1.297784000 | 3.032015000 | 2.547921000 |
| H | -1.297784000 | -3.032015000 | 2.547921000 |
| H | -1.560127000 | 5.389022000 | -1.014908000 |
| H | -1.560127000 | -5.389022000 | -1.014908000 |
| H | -1.724261000 | 1.316273000 | 2.504707000 |
| H | -1.724261000 | -1.316273000 | 2.504707000 |
| H | -2.122997000 | 5.204324000 | 0.658134000 |
| H | -2.122997000 | -5.204324000 | 0.658134000 |
| H | -2.490828000 | 2.387789000 | 3.698994000 |
| H | -2.490828000 | -2.387789000 | 3.698994000 |
| H | -3.234145000 | 0.000000000 | 1.120029000 |
| H | -3.611500000 | 6.753701000 | -0.617040000 |
| H | -3.611500000 | -6.753701000 | -0.617040000 |

| | | | |
|---|---|---|---|
| H | -3.615924000 | 3.654719000 | 1.851613000 |
| H | -3.615924000 | -3.654719000 | 1.851613000 |
| H | -3.932311000 | 5.477775000 | -1.814220000 |
| H | -3.932311000 | -5.477775000 | -1.814220000 |
| H | -4.095073000 | 1.962587000 | 1.850981000 |
| H | -4.095073000 | -1.962587000 | 1.850981000 |
| H | -4.507756000 | 5.290022000 | -0.144472000 |
| H | -4.507756000 | -5.290022000 | -0.144472000 |
| N | 2.180502000 | 1.453409000 | -1.733529000 |
| N | 2.180502000 | -1.453409000 | -1.733529000 |
| N | -2.180502000 | 1.453409000 | -1.733529000 |
| N | -2.180502000 | -1.453409000 | -1.733529000 |
| O | 0.000000000 | 1.232329000 | -3.959614000 |
| O | 0.000000000 | -1.232329000 | -3.959614000 |
| O | 1.597765000 | 3.422040000 | -2.878195000 |
| O | 1.597765000 | -3.422040000 | -2.878195000 |
| O | -1.597765000 | 3.422040000 | -2.878195000 |
| O | -1.597765000 | -3.422040000 | -2.878195000 |

**4. Cs[Pd$_2$(μ-OH)$_2$(pdp)(pdp)•], *C*$_s$, q = 0, *S* = 1/2**

| | | | |
|---|---|---|---|
| C | 2.003003000 | -3.019612000 | 2.401441000 |
| C | 2.003003000 | -3.019612000 | -2.401441000 |
| C | 3.035841000 | 1.399004000 | 2.828196000 |
| C | 3.035841000 | 1.399004000 | -2.828196000 |
| C | 3.129533000 | -0.097231000 | 4.988918000 |
| C | 3.129533000 | -0.097231000 | -4.988918000 |
| C | 3.164998000 | 0.059303000 | 3.510019000 |
| C | 3.164998000 | 0.059303000 | -3.510019000 |
| C | 3.249535000 | -0.155855000 | 1.239761000 |

| | | | |
|---|---|---|---|
| C | 3.249535000 | -0.155855000 | -1.239761000 |
| C | 3.303554000 | -0.870951000 | 2.543353000 |
| C | 3.303554000 | -0.870951000 | -2.543353000 |
| C | 3.321803000 | -0.795472000 | 0.000000000 |
| C | 3.365843000 | -2.358663000 | 2.684812000 |
| C | 3.365843000 | -2.358663000 | -2.684812000 |
| C | 4.538125000 | -0.152044000 | 5.608330000 |
| C | 4.538125000 | -0.152044000 | -5.608330000 |
| C | -0.769830000 | 0.129509000 | 2.830613000 |
| C | -0.769830000 | 0.129509000 | -2.830613000 |
| C | -1.147918000 | -1.373652000 | 4.932020000 |
| C | -1.147918000 | -1.373652000 | -4.932020000 |
| C | -1.307536000 | -1.094890000 | 3.475874000 |
| C | -1.307536000 | -1.094890000 | -3.475874000 |
| C | -1.622704000 | -4.356997000 | 2.291837000 |
| C | -1.622704000 | -4.356997000 | -2.291837000 |
| C | -1.735251000 | -1.129160000 | 1.250482000 |
| C | -1.735251000 | -1.129160000 | -1.250482000 |
| C | -1.914581000 | -1.844083000 | 2.510859000 |
| C | -1.914581000 | -1.844083000 | -2.510859000 |
| C | -2.107604000 | -1.625279000 | 0.000000000 |
| C | -2.348634000 | -0.890737000 | 5.765978000 |
| C | -2.348634000 | -0.890737000 | -5.765978000 |
| C | -2.558851000 | -3.189171000 | 2.656594000 |
| C | -2.558851000 | -3.189171000 | -2.656594000 |
| Cs | -3.663457000 | 1.113807000 | 0.000000000 |
| H | 0.546671000 | 2.320637000 | 2.135525000 |
| H | 0.546671000 | 2.320637000 | -2.135525000 |
| H | 1.248290000 | -2.651924000 | 3.100845000 |
| H | 1.248290000 | -2.651924000 | -3.100845000 |

| | | | |
|---|---|---|---|
| H | 1.656048000 | -2.783030000 | 1.391809000 |
| H | 1.656048000 | -2.783030000 | -1.391809000 |
| H | 2.074927000 | -4.109174000 | 2.499436000 |
| H | 2.074927000 | -4.109174000 | -2.499436000 |
| H | 2.576341000 | 0.749140000 | 5.409066000 |
| H | 2.576341000 | 0.749140000 | -5.409066000 |
| H | 2.585857000 | -1.014740000 | 5.244980000 |
| H | 2.585857000 | -1.014740000 | -5.244980000 |
| H | 3.444796000 | -1.869965000 | 0.000000000 |
| H | 3.685299000 | -2.611062000 | 3.701159000 |
| H | 3.685299000 | -2.611062000 | -3.701159000 |
| H | 4.120296000 | -2.772082000 | 2.003545000 |
| H | 4.120296000 | -2.772082000 | -2.003545000 |
| H | 4.472636000 | -0.260084000 | 6.697243000 |
| H | 4.472636000 | -0.260084000 | -6.697243000 |
| H | 5.090261000 | 0.765757000 | 5.381913000 |
| H | 5.090261000 | 0.765757000 | -5.381913000 |
| H | 5.103561000 | -1.000288000 | 5.206455000 |
| H | 5.103561000 | -1.000288000 | -5.206455000 |
| H | -0.244455000 | -0.862561000 | 5.282588000 |
| H | -0.244455000 | -0.862561000 | -5.282588000 |
| H | -0.747890000 | -4.364816000 | 2.948166000 |
| H | -0.747890000 | -4.364816000 | -2.948166000 |
| H | -1.009566000 | -2.450476000 | 5.091368000 |
| H | -1.009566000 | -2.450476000 | -5.091368000 |
| H | -1.267211000 | -4.263105000 | 1.261998000 |
| H | -1.267211000 | -4.263105000 | -1.261998000 |
| H | -2.146312000 | -5.315027000 | 2.395114000 |
| H | -2.146312000 | -5.315027000 | -2.395114000 |
| H | -2.191842000 | -1.106320000 | 6.829545000 |

| | | | |
|---|---|---|---|
| H | -2.191842000 | -1.106320000 | -6.829545000 |
| H | -2.481320000 | 0.189640000 | 5.646551000 |
| H | -2.481320000 | 0.189640000 | -5.646551000 |
| H | -2.606621000 | -2.587191000 | 0.000000000 |
| H | -2.893348000 | -3.312527000 | 3.692265000 |
| H | -2.893348000 | -3.312527000 | -3.692265000 |
| H | -3.268881000 | -1.390502000 | 5.442358000 |
| H | -3.268881000 | -1.390502000 | -5.442358000 |
| H | -3.458320000 | -3.245462000 | 2.029283000 |
| H | -3.458320000 | -3.245462000 | -2.029283000 |
| N | 3.089623000 | 1.165707000 | 1.430521000 |
| N | 3.089623000 | 1.165707000 | -1.430521000 |
| N | -1.101968000 | 0.070484000 | 1.465418000 |
| N | -1.101968000 | 0.070484000 | -1.465418000 |
| O | 0.478482000 | 2.756236000 | 1.264932000 |
| O | 0.478482000 | 2.756236000 | -1.264932000 |
| O | 2.905701000 | 2.484569000 | 3.356653000 |
| O | 2.905701000 | 2.484569000 | -3.356653000 |
| O | -0.130620000 | 1.028439000 | 3.386984000 |
| O | -0.130620000 | 1.028439000 | -3.386984000 |
| Pd | 2.085539000 | 2.235623000 | 0.000000000 |
| Pd | -0.429406000 | 1.363861000 | 0.000000000 |

**5. $(Cs_2)$[Pd(μ-OH)(pdp-h•)], $D_{2h}$, q = 0, *S* = 1**

| | | | |
|---|---|---|---|
| Pd | -1.666975000 | 0.000000000 | 0.000000000 |
| Pd | 1.666975000 | 0.000000000 | 0.000000000 |
| Cs | 0.000000000 | 0.000000000 | 2.901917000 |
| Cs | 0.000000000 | 0.000000000 | -2.901917000 |
| C | 2.811935000 | 2.829408000 | 0.000000000 |

| | | | |
|---|---|---|---|
| C | 2.811935000 | -2.829408000 | 0.000000000 |
| C | 4.131820000 | 3.491439000 | 0.000000000 |
| C | 4.131820000 | -3.491439000 | 0.000000000 |
| C | 4.421595000 | 1.239627000 | 0.000000000 |
| C | 4.421595000 | -1.239627000 | 0.000000000 |
| C | 5.056705000 | 0.000000000 | 0.000000000 |
| C | 5.088974000 | 2.525562000 | 0.000000000 |
| C | 5.088974000 | -2.525562000 | 0.000000000 |
| C | -2.811935000 | 2.829408000 | 0.000000000 |
| C | -2.811935000 | -2.829408000 | 0.000000000 |
| C | -4.131820000 | 3.491439000 | 0.000000000 |
| C | -4.131820000 | -3.491439000 | 0.000000000 |
| C | -4.421595000 | 1.239627000 | 0.000000000 |
| C | -4.421595000 | -1.239627000 | 0.000000000 |
| C | -5.056705000 | 0.000000000 | 0.000000000 |
| C | -5.088974000 | 2.525562000 | 0.000000000 |
| C | -5.088974000 | -2.525562000 | 0.000000000 |
| H | 0.000000000 | 2.214297000 | 0.000000000 |
| H | 0.000000000 | -2.214297000 | 0.000000000 |
| H | 4.242297000 | 4.568144000 | 0.000000000 |
| H | 4.242297000 | -4.568144000 | 0.000000000 |
| H | 6.143471000 | 0.000000000 | 0.000000000 |
| H | 6.166981000 | 2.638906000 | 0.000000000 |
| H | 6.166981000 | -2.638906000 | 0.000000000 |
| H | -4.242297000 | 4.568144000 | 0.000000000 |
| H | -4.242297000 | -4.568144000 | 0.000000000 |
| H | -6.143471000 | 0.000000000 | 0.000000000 |
| H | -6.166981000 | 2.638906000 | 0.000000000 |
| H | -6.166981000 | -2.638906000 | 0.000000000 |
| N | 3.066046000 | 1.440707000 | 0.000000000 |

| | | | |
|---|---|---|---|
| N | 3.066046000 | -1.440707000 | 0.000000000 |
| N | -3.066046000 | 1.440707000 | 0.000000000 |
| N | -3.066046000 | -1.440707000 | 0.000000000 |
| O | 0.000000000 | 1.244423000 | 0.000000000 |
| O | 0.000000000 | -1.244423000 | 0.000000000 |
| O | 1.700854000 | 3.371352000 | 0.000000000 |
| O | 1.700854000 | -3.371352000 | 0.000000000 |
| O | -1.700854000 | 3.371352000 | 0.000000000 |
| O | -1.700854000 | -3.371352000 | 0.000000000 |

**6. ($Cs_2$)[Pd(μ-OH)(pdp$^{\bullet}$)], $C_{2v}$, q = 0, $S$ = 1**

| | | | |
|---|---|---|---|
| Pd | 0.000000000 | 1.394806000 | 1.361517000 |
| Pd | 0.000000000 | -1.394806000 | 1.361517000 |
| Cs | 2.414151000 | 0.000000000 | -1.710360000 |
| Cs | -2.414151000 | 0.000000000 | -1.710360000 |
| C | 0.000000000 | 3.543969000 | -1.281217000 |
| C | 0.000000000 | -3.543969000 | -1.281217000 |
| C | 1.247915000 | 3.212186000 | -0.755452000 |
| C | 1.247915000 | -3.212186000 | -0.755452000 |
| C | 2.428199000 | 3.514042000 | -3.899266000 |
| C | 2.428199000 | -3.514042000 | -3.899266000 |
| C | 2.515783000 | 3.600660000 | -1.362609000 |
| C | 2.515783000 | -3.600660000 | -1.362609000 |
| C | 2.661355000 | 4.370560000 | -2.639549000 |
| C | 2.661355000 | -4.370560000 | -2.639549000 |
| C | 2.855377000 | 2.340396000 | 0.533195000 |
| C | 2.855377000 | -2.340396000 | 0.533195000 |
| C | 3.505369000 | 3.078949000 | -0.580811000 |
| C | 3.505369000 | -3.078949000 | -0.580811000 |

| | | | |
|---|---|---|---|
| C | 4.985443000 | 3.144302000 | -0.745721000 |
| C | 4.985443000 | -3.144302000 | -0.745721000 |
| C | 5.597584000 | 4.430215000 | -0.161676000 |
| C | 5.597584000 | -4.430215000 | -0.161676000 |
| C | -1.247915000 | 3.212186000 | -0.755452000 |
| C | -1.247915000 | -3.212186000 | -0.755452000 |
| C | -2.428199000 | 3.514042000 | -3.899266000 |
| C | -2.428199000 | -3.514042000 | -3.899266000 |
| C | -2.515783000 | 3.600660000 | -1.362609000 |
| C | -2.515783000 | -3.600660000 | -1.362609000 |
| C | -2.661355000 | 4.370560000 | -2.639549000 |
| C | -2.661355000 | -4.370560000 | -2.639549000 |
| C | -2.855377000 | 2.340396000 | 0.533195000 |
| C | -2.855377000 | -2.340396000 | 0.533195000 |
| C | -3.505369000 | 3.078949000 | -0.580811000 |
| C | -3.505369000 | -3.078949000 | -0.580811000 |
| C | -4.985443000 | 3.144302000 | -0.745721000 |
| C | -4.985443000 | -3.144302000 | -0.745721000 |
| C | -5.597584000 | 4.430215000 | -0.161676000 |
| C | -5.597584000 | -4.430215000 | -0.161676000 |
| H | 0.000000000 | 4.145723000 | -2.182087000 |
| H | 0.000000000 | -4.145723000 | -2.182087000 |
| H | 1.435090000 | 3.051726000 | -3.878951000 |
| H | 1.435090000 | -3.051726000 | -3.878951000 |
| H | 1.958636000 | 5.212725000 | -2.652048000 |
| H | 1.958636000 | -5.212725000 | -2.652048000 |
| H | 2.156677000 | 0.000000000 | 1.985296000 |
| H | 2.501438000 | 4.124314000 | -4.807414000 |
| H | 2.501438000 | -4.124314000 | -4.807414000 |
| H | 3.183257000 | 2.721069000 | -3.971048000 |

| | | | |
|---|---|---|---|
| H | 3.183257000 | -2.721069000 | -3.971048000 |
| H | 3.666669000 | 4.800912000 | -2.688655000 |
| H | 3.666669000 | -4.800912000 | -2.688655000 |
| H | 5.183935000 | 5.314686000 | -0.658918000 |
| H | 5.183935000 | -5.314686000 | -0.658918000 |
| H | 5.249003000 | 3.077581000 | -1.810731000 |
| H | 5.249003000 | -3.077581000 | -1.810731000 |
| H | 5.374099000 | 4.501611000 | 0.907285000 |
| H | 5.374099000 | -4.501611000 | 0.907285000 |
| H | 5.421786000 | 2.278679000 | -0.232019000 |
| H | 5.421786000 | -2.278679000 | -0.232019000 |
| H | 6.686137000 | 4.432358000 | -0.293053000 |
| H | 6.686137000 | -4.432358000 | -0.293053000 |
| H | -1.435090000 | 3.051726000 | -3.878951000 |
| H | -1.435090000 | -3.051726000 | -3.878951000 |
| H | -1.958636000 | 5.212725000 | -2.652048000 |
| H | -1.958636000 | -5.212725000 | -2.652048000 |
| H | -2.156677000 | 0.000000000 | 1.985296000 |
| H | -2.501438000 | 4.124314000 | -4.807414000 |
| H | -2.501438000 | -4.124314000 | -4.807414000 |
| H | -3.183257000 | 2.721069000 | -3.971048000 |
| H | -3.183257000 | -2.721069000 | -3.971048000 |
| H | -3.666669000 | 4.800912000 | -2.688655000 |
| H | -3.666669000 | -4.800912000 | -2.688655000 |
| H | -5.183935000 | 5.314686000 | -0.658918000 |
| H | -5.183935000 | -5.314686000 | -0.658918000 |
| H | -5.249003000 | 3.077581000 | -1.810731000 |
| H | -5.249003000 | -3.077581000 | -1.810731000 |
| H | -5.374099000 | 4.501611000 | 0.907285000 |
| H | -5.374099000 | -4.501611000 | 0.907285000 |

| | | | |
|---|---|---|---|
| H | -5.421786000 | 2.278679000 | -0.232019000 |
| H | -5.421786000 | -2.278679000 | -0.232019000 |
| H | -6.686137000 | 4.432358000 | -0.293053000 |
| H | -6.686137000 | -4.432358000 | -0.293053000 |
| N | 1.467307000 | 2.456047000 | 0.365946000 |
| N | 1.467307000 | -2.456047000 | 0.365946000 |
| N | -1.467307000 | 2.456047000 | 0.365946000 |
| N | -1.467307000 | -2.456047000 | 0.365946000 |
| O | 1.236301000 | 0.000000000 | 2.297031000 |
| O | 3.437971000 | 1.657861000 | 1.384304000 |
| O | 3.437971000 | -1.657861000 | 1.384304000 |
| O | -1.236301000 | 0.000000000 | 2.297031000 |
| O | -3.437971000 | 1.657861000 | 1.384304000 |
| O | -3.437971000 | -1.657861000 | 1.384304000 |
